# Automatic adventitious respiratory sound analysis: A systematic review

**DOI:** 10.1371/journal.pone.0177926

**Published:** 2017-05-26

**Authors:** Renard Xaviero Adhi Pramono, Stuart Bowyer, Esther Rodriguez-Villegas

**Affiliations:** Department of Electrical and Electronic Engineering, Imperial College London, London, United Kingdom; Charité - Universitätsmedizin Berlin, GERMANY

## Abstract

**Background:**

Automatic detection or classification of adventitious sounds is useful to assist physicians in diagnosing or monitoring diseases such as asthma, Chronic Obstructive Pulmonary Disease (COPD), and pneumonia. While computerised respiratory sound analysis, specifically for the detection or classification of adventitious sounds, has recently been the focus of an increasing number of studies, a standardised approach and comparison has not been well established.

**Objective:**

To provide a review of existing algorithms for the detection or classification of adventitious respiratory sounds. This systematic review provides a complete summary of methods used in the literature to give a baseline for future works.

**Data sources:**

A systematic review of English articles published between 1938 and 2016, searched using the Scopus (1938-2016) and IEEExplore (1984-2016) databases. Additional articles were further obtained by references listed in the articles found. Search terms included adventitious sound detection, adventitious sound classification, abnormal respiratory sound detection, abnormal respiratory sound classification, wheeze detection, wheeze classification, crackle detection, crackle classification, rhonchi detection, rhonchi classification, stridor detection, stridor classification, pleural rub detection, pleural rub classification, squawk detection, and squawk classification.

**Study selection:**

Only articles were included that focused on adventitious sound detection or classification, based on respiratory sounds, with performance reported and sufficient information provided to be approximately repeated.

**Data extraction:**

Investigators extracted data about the adventitious sound type analysed, approach and level of analysis, instrumentation or data source, location of sensor, amount of data obtained, data management, features, methods, and performance achieved.

**Data synthesis:**

A total of 77 reports from the literature were included in this review. 55 (71.43%) of the studies focused on wheeze, 40 (51.95%) on crackle, 9 (11.69%) on stridor, 9 (11.69%) on rhonchi, and 18 (23.38%) on other sounds such as pleural rub, squawk, as well as the pathology. Instrumentation used to collect data included microphones, stethoscopes, and accelerometers. Several references obtained data from online repositories or book audio CD companions. Detection or classification methods used varied from empirically determined thresholds to more complex machine learning techniques. Performance reported in the surveyed works were converted to accuracy measures for data synthesis.

**Limitations:**

Direct comparison of the performance of surveyed works cannot be performed as the input data used by each was different. A standard validation method has not been established, resulting in different works using different methods and performance measure definitions.

**Conclusion:**

A review of the literature was performed to summarise different analysis approaches, features, and methods used for the analysis. The performance of recent studies showed a high agreement with conventional non-automatic identification. This suggests that automated adventitious sound detection or classification is a promising solution to overcome the limitations of conventional auscultation and to assist in the monitoring of relevant diseases.

## Introduction

Most diseases related to an obstructed or restricted respiratory system can be characterised from the sounds generated while breathing. These include asthma, COPD, and pneumonia amongst others. Airway abnormalities can cause breathing sounds to be abnormal. Examples of this could be the absence of sounds or additive unusual ones. The latter are referred to as adventitious sounds. An expert can perform auscultation using a stethoscope to detect abnormalities in sounds and use this information when making a diagnosis. However, the correct detection of these sounds relies on both, the presence of an “expert”, and their degree of expertise.

While computerised respiratory sound analysis, specifically for the detection or classification of adventitious sounds, has been the focus of an increasing number of studies recently, a standardised approach and comparison has not been well established. Several reviews related to automatic adventitious sounds analysis have been published [1–6]. The article in [1] provided a review of 49 articles which included the type of sensor, the data set, the features, the analysis techniques, and also the performance metrics used. The review categorised features into time-domain, frequency-domain, wavelet-domain, and a combination of different domains. Signal pre-processing techniques such as de-noising, resampling, and analogue pre-filtering were also presented, as well as the number of sensors and their positioning. Information on analysis type, approach, and data management was not reviewed. The conclusion of this work was that a multi domain feature has advantages in characterising different types of lung sounds.

A review of computerised respiratory sounds specifically in patients with COPD was done in [2]. This included a total of seven papers. The focus of this review was studies that tried to find the characteristics of adventitious sounds in COPD (wheeze, crackle, and rhonchi), including occurrence timing and the power spectrum.

The review in [3] provided information on machine learning techniques used in lung sound analysis. This covered types of analysis, sensor type, number of subjects, machine learning techniques used, and the outcome of each reference. A total of 34 studies were reviewed. The review concluded that artificial intelligence techniques are needed to improve accuracy and enable commercialisation as a product. Another review, published by the same group, provided a summary of 55 studies on computer-based respiratory sound analysis [4]. The review included analysis type, sensor used, data set used, sensor location, and method of analysis. This work provided several recommendations for sensor type, position, and the use of more advanced machine learning techniques.

The survey in [5] focused on automated wheeze detection for asthmatic patients, and provided a review on instrumentation, placement, processing methods, features used, and the outcome, of a total of 27 studies. The study recommended placing the stethoscope on the trachea as this preserves more frequency information when compared to the chest wall.

A systematic review and meta-analysis of computerised lung sound analysis to aid in the diagnosis of diseases was presented in [6]. A total of 8 articles were selected for this systematic review which consisted of studies on wheeze, crackle, and other adventitious sounds for specific diseases such as asthma and COPD. The review included the number of subjects, age range, gender ratio, methodology, case, recording device, algorithm, and type of sounds analysed. The quality of each study was assessed using the Newcastle-Ottawa Score (NOS). The NOS is normally used for assessing non-randomised studies including control-studies. Four of the selected articles were then used for meta-analysis. This obtained an average of 80% sensitivity and 85% specificity in abnormal sound detection.

This systematic review adds to these existing reviews by providing more thorough information in a standardised format, with more works being reviewed, and more recent developments included. The comparison of this work with the previously mentioned reviews can be seen below.

- [1], 2015, 49 articles, focused on respiratory sound analysis
- [2], 2015, 7 articles, focused on COPD patients
- [3], 2013, 34 articles, focused on machine learning techniques in lung sound analysis
- [4], 2013, 55 articles, focused on computer-based respiratory sound analysis
- [5], 2012, 21 articles, focused on wheeze analysis for asthma patients
- [6], 2011, 8 articles, focused on abnormal lung sound detection
- This work, 77 articles, focused on automatic adventitious respiratory sound analysis.

**Fig 1:**
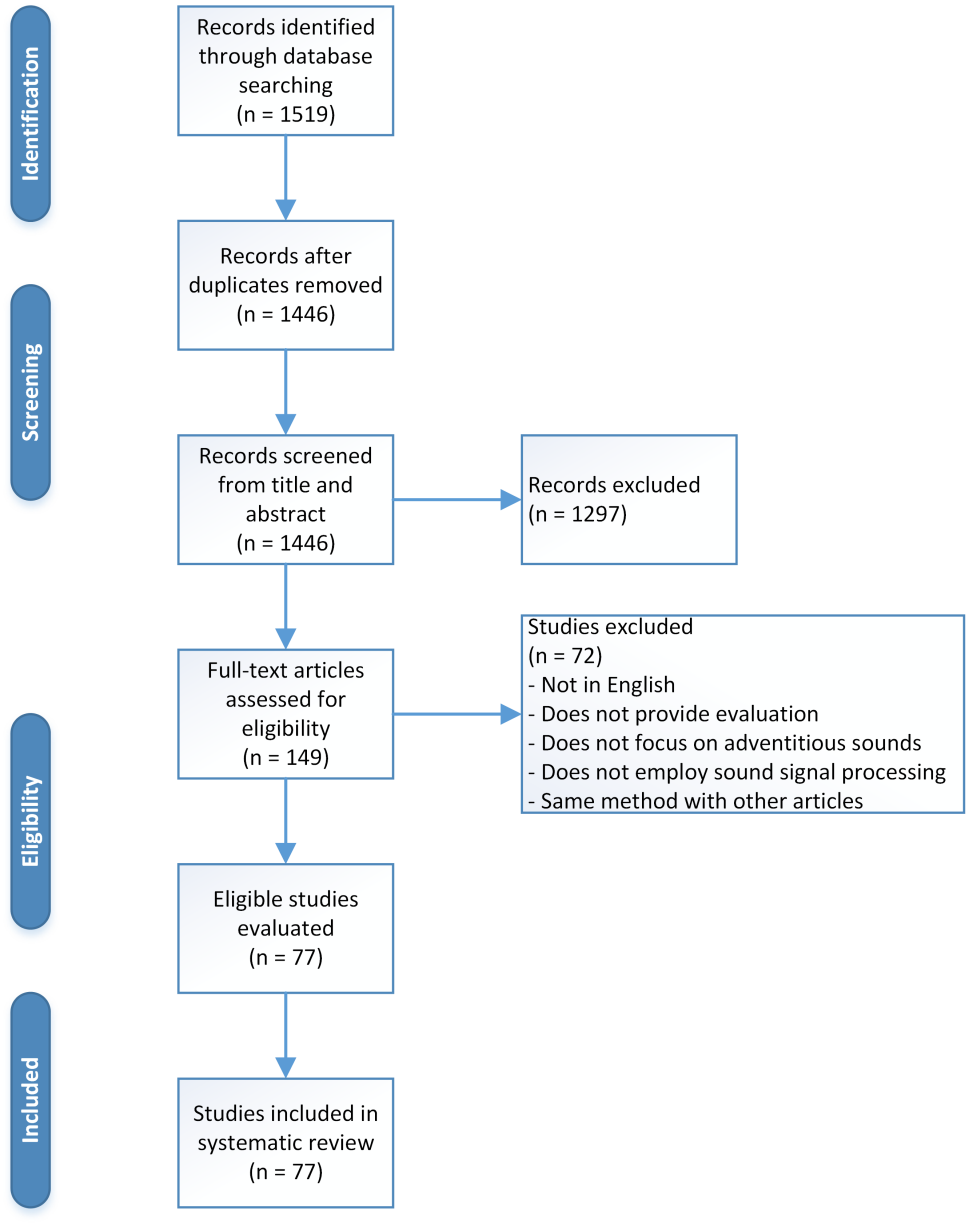
Study selection flow diagram.

A standardised approach was used for this systematic review (Fig 1 and S1 File). Table 1 provides a summary of normal breath sounds, while adventitious sounds are summarised in Table 2. Analysis type, level, and approach to differentiate between studies are provided in Table 3. Dataset size and data management, which are an important part in the analysis, are stated in Tables 4 and 5. Furthermore, performance analysis of several studies with the same approach and purpose is given in Table 6. A discussion based on the outcome of the review is provided, as well as recommendations for future works.

**Table 1:** Normal breath sounds.

Breath Sounds	Location	Range^a^	Pitch^b^	Quality^c^	Timing (I:E ratio)^d^	Pause^e^
Vesicular	Most of lung fields	100—1,000 Hz Energy drop at 200 Hz	Low	Low-pass filtered noise like Soft Rustling sound	During inspiration and early expiration (2:1 ratio)	Pause between different breath cycle
Broncho-Vesicular	Between scapulae on posterior chest and center part of anterior chest	Intermediate between Vesicular and Bronchial	Intermediate	Intermediate intensity	During both inspiration and expiration (1:1 ratio)	N/M
Bronchial	Large airways on chest near second and third intercostal space	Similar to Tracheal	High	Loud Hollow	During both inspiration and expiration (1:2 ratio)	Short pause between inspiration and expiration phase
Tracheal	Suprasternal notch on trachea	100—5,000 Hz Energy drop at 800 Hz	High	Harsh Very loud	During both inspiration and expiration (1:1 ratio)	Distinct pause between inspiration and expiration phase
Mouth	Mouth	200—2,000 Hz	N/M	White-noise like Silent when normal	N/M	N/M

^a^Information from [8–10, 12, 13]

^b^Information from [8, 9]

^c^Information from [8, 13]

^d^Information from [8, 11]

^e^Information from [8]

Abbreviation N/M: Not Mentioned in [8–13]

**Table 2:** Types of adventitious sounds and its characteristics.

Types	Continuity	Duration^a^	Timing^b^	Pitch^c^	Quality^d^	Cause^e^	Disease Associated^f^
Wheeze	Continuous	> 80*ms*	Inspiratory, Mostly Expiratory, Biphasic	High (> 400*Hz*)	Sibilant, Musical	Airway narrowing, airflow limitation	Asthma, COPD, Foreign body
Rhonchi	Continuous	> 80*ms*	Inspiratory, Mostly Expiratory, Biphasic	Low (< 200*Hz*)	Sibilant, Musical	Secretion in bronchial, muchosal thickening	Bronchitis, COPD
Stridor	Continuous	> 250*ms*	Mostly Inspiratory, Expiratory, Both	High (> 500*Hz*)	Sibilant, Musical	Turbulent airflow in larynx or lower bronchial tree (Upper airway obstruction)	Epiglottitis, foreign body, croup, laryngeal oedema
Fine Crackle	Discontinuous	± 5 ms	Inspiratory (late)	High (650 Hz)	Non-musical, Explosive	Explosive opening of small airways	Pneumonia, Congestive heart failure, Lung fibrosis
Coarse Crackle	Discontinuous	± 15 ms	Mostly Inspiratory (early), Expiratory, Both	Low (350 Hz)	Non-musical, Explosive	Air bubble in large bronchi or bronchiectatic segments	Chronic bronchitis, bronchiectasis, COPD
Pleural Rub	Discontinuous	> 15*ms*	Biphasic	Low (< 350*Hz*)	Non-Musical, Rhythmic	Pleural membrane rubbing against each other	Inflammation of lung membrane, lung tumour
Squawk	Continuous	± 200 ms	Inspiratory	Low (200—300 Hz)	Short Musical and non-musical	Oscillation of peripheral airways	Hypersensitivity pneumonia, pneumonia
Gasp	Continuous	> 250*ms*	Inspiratory	High	Whoop	Gasping for breath	Whooping cough

^a^Information from [10, 17, 19, 20, 23]

^b^Information from [8, 10, 20, 21]

^c^Information from [10, 14, 20, 23]

^d^Information from [8, 10, 18–20]

^e^Information from [8, 15, 16, 19–21, 24]

^f^Information from [8, 10, 20, 22]

**Table 3:** Sound and analysis type.

Ref	Year	Sound Type							Approach		Level		
		W	R	C	S	E	U	SC	De	Cl	Se	Ev	Re
[37]	2016	•	o	o					•	•	•		•
[38]	2016	•		•						•			•
[39]	2016	•							•	•		•	•
[40]	2016	•							•		•		
[41]	2016	•	•							•	•	•	
[42]	2016	o		•					•		•		
[43]	2016						•			•	•	•	•
[44]	2016	•		•						•		•	
[45]	2016	•		•						•	•	•	
[46]	2016	•		•						•		•	
[47]	2015	•		o					•	•		•	•
[48]	2015	•	•	•					•		•		
[49]	2015	•							•	•		•	•
[50]	2015	•		•	•					•	•		•
[51]	2015			•						•			•
[52]	2015	•	•	•						•	•	•	•
[53]	2015	•			o				•		•		
[54]	2015	•		o					•		•	•	•
[55]	2015	•		•	•	•				•		•	
[56]	2015			•					•	•	•	•	
[57]	2015	o		o				•		•	•		•
[58]	2015	•							•		•		
[59]	2015	•								•		•	
[60]	2015	•	•	•						•	•	•	•
[61]	2015	•								•	•		
[62]	2015			•						•		•	
[63]	2014	•			•					•		•	
[64]	2014		•	•						•			•
[65]	2014	•	•		•					•		•	
[66]	2014	•							•	•			•
[67]	2014			•					•		•		
[68]	2014	•		•		•				•		•	
[69]	2014	•		•						•	•	•	
[70]	2014							•		•			•
[71]	2014	•		•						•		•	
[72]	2013	•		•						•			•
[73]	2013	•							•		•	•	•
[74]	2013	•							•	•		•	•
[75]	2013							•		•			•
[76]	2013			•						•	•		
[77]	2013	•							•		•		•
[78]	2012	•		•						•			•
[79]	2012	•	•	•		•				•	•	•	•
[80]	2012						•			•			•
[81]	2012	•								•	•		
[82]	2012			•					•		•	•	
[83]	2012	•								•	•	•	
[84]	2012			•					•		•		
[85]	2012						•			•		•	
[86]	2011	•			•					•		•	
[87]	2010	•		•	•					•			•
[88]	2010	•								•		•	
[89]	2009	•								•	•		
[90]	2009	•							•		•		
[91]	2009	•		•	•	•				•	•	•	
[92]	2009			•		•				•	•		
[93]	2009	•	•	•		•	•			•	•	•	
[94]	2009	•								•		•	
[95]	2009	•								•	•		•
[96]	2008							•		•	•	•	•
[97]	2008	•							•			•	•
[98]	2008	•								•		•	
[99]	2007	•								•	•		
[100]	2007	o		•					•	•	•	•	
[101]	2007	•							•			•	•
[102]	2005							•		•	•	•	
[103]	2005	•							•				•
[104]	2005			•						•	•		
[105]	2005			•						•		•	
[106]	2004	•							•		•	•	
[107]	2000	•		•	•	•				•		•	
[108]	1997			•					•	•	•		
[109]	1997			•				•		•	•	•	•
[110]	1996	•		•					•		•		
[111]	1995	•								•	•		
[112]	1992			•					•	•	•	•	
[113]	1984							•		•		•	

Symbol ‘•’ denotes focus of study in corresponding article

while ‘o’ denotes sound included in study but not as main focus

W: Wheeze, R: Rhonchi, C: Crackle, S: Stridor

E: Egophony, Squawk, or Pleural Rub

U: Unspecified CAS or DAS, SC: Sound Cause

De: Detection, Cl: Classification

Se: Segment, Ev: Event, Re: Recording

**Table 4:** Sensor and data source for lung sound analysis.

Ref	Year	Data Source		# Sensor	Sensor Position					Total Data
		Sensor	Database		Neck	Anterior	Posterior	Mouth	Multiple	
[37]	2016	[114]★	-	1				•		95 recordings
[38]	2016	custom♦	-	1	•	•	•		•	227 recordings 171 normal 33 wheeze 19 crackle 4 w&c
[39]	2016	[122]♦	ATS, COPD	1	N/M					112 recordings 70 wheeze 42 normal
[40]	2016	[115]★ [137]♦	-	1	•					18 volunteers 9 asthmatic 9 normal 3036 segments 568 wheeze 2468 normal
[41]	2016	[116]★	-	5	•		•		•	870 events 1494 segments
[42]	2016	[122]♦	-	1		•	•		•	20 volunteers 40 recordings 400 crackle events
[43]	2016	Electronic ♦	-	1		•	•		•	3120 short recordings 1560 normal 1560 abnormal
[44]	2016	-	[129]	N/A						36 recordings 318 events
[45]	2016	-	[124] [138] [139]	N/A						30 volunteers 72 events
[46]	2016	[140]★	-	14		•			•	600 events 200 crackle 200 normal 200 wheeze
[47]	2015	custom ♦	-	1	•	•	•		•	38 patients 57 recording 28 normal 26 wheeze 3 crackle
[48]	2015	Condenser★ analog ♦	[128]	1		•				20 recordings and additional data
[49]	2015	[115]★ [137]♦	-	1	•					58 recordings
[50]	2015	N/M	[127]	N/M						45 recordings
[51]	2015	N/M								41 recordings
[52]	2015	Piezoelectric★ electronic ♦	-	1		•	•		•	130 recordings 66 patient 64 healthy 3140 events 1567 abnormal 1573 normal
[53]	2015	[123]■	-	1			•			45 recordings
[54]	2015	[121]♦	-	1		•	•		•	12 volunteers 113 wheeze events
[55]	2015	-	[129] [130]	N/A						28 recordings
[56]	2015	[119]♦ [120]♦	-	1	N/M					24 recordings
[57]	2015	[140]★	-	14			•		•	40 recordings 20 healthy 10 obstructive 10 restrictive
[58]	2015	-	[124]	N/A						26 recordings 17 wheeze 9 normal 1188 segments 898 normal 290 wheeze
[59]	2015	[140]★	-	14			•		•	7 volunteers 231 events
[60]	2015	Piezoelectric★ electronic ♦	-	1		•			•	230 recordings 115 normal 115 abnormal
[61]	2015	[117]★	[125] [126]	3	•	•			•	260 segments
[62]	2015	[121]♦	-	1		•	•		•	100 events
[63]	2014	-	[130] [131] [133]	N/A						9 recordings Total Data N/M
[64]	2014	[120]♦	-	1			•		•	60 volunteers
[65]	2014	[140]★	[129] [131] [132]	1	•					339 events 239 events
[66]	2014	N/M								371 recordings
[67]	2014	-	[124]	N/A						2 recordings
[68]	2014	★	[138] [139]	1	•					30 recordings 120 events
[69]	2014	-	[124]	N/A						13 events
[70]	2014	-	[124]	N/A						68 recordings
[71]	2014	-	[129]	N/A						92 events 27 normal 31 crackle 34 wheeze
[72]	2013	★	-	7	•	•	•		•	60 volunteers 345 recordings
[73]	2013	N/M								6 events
[74]	2013	[119]♦	-	1	•	•	•		•	40 recordings
[75]	2013	-	[124]	N/A						68 recordings
[76]	2013	[140]★	-	14			•		•	26 volunteers 6000 segments
[77]	2013	soft ♦	-	1	•					8 volunteers 59 recordings
[78]	2012	[119]♦	-	1		•			•	28 recordings
[79]	2012	Piezoelectric★ electronic ♦	-	1	•					126 recordings 63 normal 63 abnormal 723 events 351 normal 372 abnormal
[80]	2012	-	[124]	N/A						47 recordings
[81]	2012	N/M								180 segments 98 normal 82 wheezes
[82]	2012	-	ACCP	N/A						10 short recordings (200ms) 33 crackle events
[83]	2012	N/M								26 recordings
[84]	2012	N/M								433 segments
[85]	2012	[140]★	[129] [131] [132]	1	•					47 recordings 689 events
[86]	2011	[140]★	[129] [131] [132]	1	•					585 events
[87]	2010	-	[124]	N/A						4-7 recordings each class
[88]	2010	[140]★	-	5	•		•		•	21 volunteers 393 wheeze events
[89]	2009	[140]★	-	14			•		•	7 volunteers 492 segments
[90]	2009	-	[124]	N/A						24 recordings 2807 segments
[91]	2009	Electronic ♦	-	1			•			36 recordings 360 events
[92]	2009	-	[129] [132] [134] [135]	N/A						25 recordings 9 FC 8 CC 8 Squawk 96 segments 32 FC 32 CC 32 squawk
[93]	2009	Condenser★ piezoelectric ♦	-	1		•	•		•	162 volunteers 1544 events
[94]	2009	-	[124]	N/A						40 events 28 recordings 112 events
[95]	2009	-	[129]	N/A						17 recordings
[96]	2008	[140]★	-	1			•			65 volunteers
[97]	2008	-	[125]	N/A						40 events 21 normal 19 wheeze
[98]	2008	[141]★	[129] [132]	1	•					14 volunters 186 events 100 normal 86 wheeze
[99]	2007	ECM★ [137]♦	-	1	•					30 volunteers
[100]	2007	-	[129] [131] [142]	N/A						18 recordings 5 FC 5 CC 4 normal 4 wheeze 182 crackle events
[101]	2007	[141]★	-	5	•	•	•		•	13 volunteers 422 wheeze events
[102]	2005	Electret★	-	2		•			•	57 volunteers 18 Obstructive 19 Restrictive 20 healthy
[103]	2005	[115]★	-	1	•					16 volunteers 12 asthmatic 4 healthy
[104]	2005	★	-	25			•		•	29 volunteers 10 healthy 19 patients
[105]	2005	N/M								2 volunteers 391 events 238 CC 153 FC
[106]	2004	Piezoelectric■	-	1	•					31 volunteers 16 asthmatic 15 healthy
[107]	2000	LS-60★	[129] [131]	2		•			•	2127+321 events 788+251 abnormal 1360+70 normal
[108]	1997	-	ACCP	N/A						2 recordings
[109]	1997	★	-	2		•			•	69 volunteers 28 obstructive 23 restrictive 18 healthy
[110]	1996	N/M				•				13 volunteers 4 healthy 9 patients 5000 segments
[111]	1995	N/M								710 segments 375 wheeze 335 normal
[112]	1992	★	-	1	N/M					9 patients
[113]	1984	-	[136]	N/A						147 events

^★^ denotes microphone,

^♦^ denotes stethoscope,

^■^ denotes accelerometer

ATS: American Thoracic Society website, COPD: COPD website

ACCP: American College Chest Physician teaching tape

N/A: Not Applicable, N/M: Not Mentioned

**Table 5:** Data, features, and methods of analysis.

Ref	Year	Data Set				Features	Method	Performance
		Training	Validation	Test	Total			
[37]	2016	70 Rec, 20 W, 50 N	25 Rec, 7 W, 18 N	39 Rec, 10 W, 29 N	95 Rec	Spectral features (PSD mean, harmonics)	SVM, LRM	71.4% Se, 88.9% Sp, for SVM on validation set at Rec level
[38]	2016	5-fold CV			227 Rec	Denoising autoencoders	SVM	90% Se, 64% Sp for W Rec level and 90% Se, 44% Sp for C Rec level
[39]	2016	N/A		112 Rec	112 Rec	Rule-based Seg selection, Power Ratio	Threshold	90% Se, 90.48% Sp at Rec level
[40]	2016	N/M			3036 Seg	MFCC	GMM	88.1% Se, 99.5% Sp at Seg level
[41]	2016	65%	10-fold CV	35%	870 Ev	Ensemble Empirical Mode Decomposition and Instantaneous Frequency	SVM	94.2% Se, 96.1% Sp, for SVM on best iteration of test set at Ev level
[42]	2016	10-fold CV		LOOCV	400 Ev	Musical features, wavelet-based, teager energy, entropy	LRM	76 ± 23% Se, 77 ± 22% PPV at Seg level
[43]	2016	LOOCV			3120 Rec	MFCC	HMM	Best Acc at Seg level 82.82%, average Acc of 87.7% at Rec level
[44]	2016	219 Ev, 71 N, 39 FC, 39 CC, 35 mono W, 35 poly W	40 holdout CV	99 Ev, 31 N, 18 FC, 18 CC, 16 mono W, 16 poly W	318 Ev	Higher Order Statistics (Cumulants)	GA + k-NN and NB	94.6% Overall Acc on test set at Ev level
[45]	2016	LOOCV			72 Ev	LFCC, MFCC, IMFCC, and LPCC	MLP	97.83% best Overall Acc using MFCC at Ev level
[46]	2016	LOOCV			600 Ev	Energy of High Q-Factor Wavelet coefficients	k-NN, SVM	95.17% average Acc for SVM at Ev level
[47]	2015	LOOCV			57 Rec	Peak to mean ratio, expected number of false positives	Threshold+SVM	86% Acc on Rec level
[48]	2015	20 Rec	-	Multiple sets	> 20 Rec	13 MFCC each with first and second derivatives	k-NN	Performance of 6 different types of test reported as Acc
[49]	2015	23 Rec, 13 W, 10 N	-	35 Rec, 19 W, 16 N	58 Rec	Duration, frequency range, area, power, and slope of spectrum	BPNN	94.6% Se, 100% Sp at Rec level
[50]	2015	N/A		45 Rec	45 Rec	Entropy-based Features	Threshold	99% Acc Stridor, 70% Acc W, 87% Acc C, 99% Acc N, at Rec level
[51]	2015	41 Rec			41 Rec	Spectral features	GMM	92.85% Se, 100% Sp at Rec level
[52]	2015	LOOCV			130 Rec	MFCC, correlation score with other auscultation point and other Seg	HMM	Best Acc of 92.26% at Ev level and best Acc of 91% at Rec level
[53]	2015	21 Rec, 5 W, 21 Non-W	20%-80% Train Validation Set repeated 20 times	Leave-one-out CV	45 Rec	MFCC, Kurtosis, Entropy	2 SVM + Threshold	97.68% Reliability (TPR.TNR) using MFCC at Seg level
[54]	2015	10-fold CV			113 Ev	Musical features and spectrogram signature	LRM, RF	90.9% ± 2% Se, 99.4% ± 1% Sp for RF at Seg level
[55]	2015	70% of data	15% of data	15% of data	28 Rec	Averaged Power Spectrum	ANN	97.8% Se, 100% Sp on test set at Ev level
[56]	2015	N/A		24 Rec	24 Rec	Fractal Dimension, CORSA criterion for Crackle	Threshold	Average Se of 89 ± 10%, PPV of 95 ± 11% at Ev level for different Rec
[57]	2015	LOOCV			40 Rec	AR Model	GMM, SVM	90% best total Acc for GMM on Rec level
[58]	2015	LTOCV			1188 Seg	MFCC, WPT, FT	C-Weighted SVM	81.5 ± 10% Se, 82.6 ± 7% Sp for MFCC features on Seg level
[59]	2015	N/M			231 Ev	Quartile Frequency Ratios, Mean Crossing Irregularity	SVM, k-NN, NB	75.78% best Overall Acc for kNN at Ev level
[60]	2015	LOOCV			230 Rec	MFCC	Subject adaptation HMM	89.4% Se, 80.9% Sp at Ev level and 90.4% Se, 78.3% Sp at Rec level
[61]	2015	10-fold CV			260 Seg	Audio Spectral Envelope and Tonality Index	SVM	93% Overall Acc at Seg level
[62]	2015	N/A		100 Ev, 50 C, 50 N	100 Ev	Mathematical morphology	Threshold	86% Se, 92% Sp at Ev level
[63]	2014	N/M				Delay Coordinate	Threshold	98.39% Acc at Ev level
[64]	2014	5-fold CV			60 Vol	frequency ratio, average instantaneous frequency, eigenvalues	SVM	Individual Acc reported for all case of one-versus-one and one-versus-all for all features at Rec level
[65]	2014	LOOCV			578 Ev	Instantaneous Kurtosis, Discriminanting Function, Sample Entropy	SVM	97.7% Mean Acc (Inhale), 98.8% Mean Acc (exhale) at Ev level
[66]	2014	371 Ev			371 Rec	Centroid, time duration, slope, and area ratio of spectrum	SVM	88.7% Se, 93.9% Sp at Rec level
[67]	2014	LOOCV			2 Rec	Teager energy, wavelet, fractal dimension, empirical mode decomposition, entropy, and GARCH process	LRM	MCC of 80% at Seg level
[68]	2014	5-fold CV			120 Ev	Lacunarity, sample entropy, skewness, and kurtosis	SVM, ELM	86.30% Se, 86.90% Sp for ELM at Ev level
[69]	2014	LOOCV			13 Ev	MFCC	MLP	100% Acc W, 75% Acc C, 80% Acc N at Ev level
[70]	2014	10-fold CV			68 Rec	MFCC	SVM, k-NN	100% Acc N, 100% Acc AOP, 96% Acc PP for kNN at Rec level
[71]	2014	60 Ev	14 Ev	18 Ev	92 Ev	Wavelet packet transform	ANN	98.89% best average Acc for Symlet-10 wavelet base at Ev level on test set
[72]	2013	75%-25% Train Validation Set repeated 6 times			345 Rec	Spectrogram evaluation for W, db5 Wavelet degree of similarity for C	ANN	80% Se, 67% Sp at Rec level
[73]	2013	N/A		6 Ev	6 Ev	Time Frequency Analysis and Wavelet Packet Decomposition	Threshold	All Ws detected
[74]	2013	N/A		40 Rec	40 Rec	Time Frequency Analysis	Threshold	99.2% Se, 72.5% Sp at Ev level
[75]	2013	60%-40% Train Validation Set repeated 25 times			68 Rec	MFCC	SVM	94.11% Acc N, 92.31% Acc AOP, 88% Accruacy PP, for SVM at Rec level
[76]	2013	2000 Seg, 1000 N, 1000 C	2000 Seg, 1000 N, 1000 C	2000 Seg, 1000 N, 1000 C	6000 Seg	Time Frequency Analysis (Spectrogram), Time Scale Analysis (Wavelet)	SVM, MLP, k-NN	97.5% Overall Acc rate for SVM using Time Frequency Analysis at Seg level
[77]	2013	N/A		59 Rec	59 Rec	Correlation Coefficient	Threshold	88% Se, 94% Sp at Rec level
[78]	2012	10-fold CV			28 Rec	Cortical Model	SVM	89.44% Se, 80.50% Sp at Rec level
[79]	2012	LOOCV			126 Rec, 723 Ev	Power, spectral features, and duration distribution	HMM	88.7% Se, 91.5% Sp at Ev level and 87% Se, 81% Sp at Rec level
[80]	2012	N/A		47 Rec	47 Rec	Local similarity measure using Mutual Information, Weighted cepstral features	Threshold	High Acc for local similarity measure and separability index of 1 for weighted cepstral
[81]	2012	N/A		180 Seg	180 Seg	fractional Hilbert transform	Threshold	Acc of 90.5% at Seg level
[82]	2012	N/A		33 C Ev	33 Ev	fractional Hilbert transform and correlation coefficient	Threshold	Se 94.28%, PPV 97.05% at Ev level
[83]	2012	N/A		26 Rec, 13 N, 13 W	26 Rec	LPC prediction error ratio	Threshold	70.9% Se, 98.6% Sp at Ev level
[84]	2012	N/A		433 Seg	433 Seg	Abnormality level	Threshold	84.5% Acc at Seg level
[85]	2012	50%-50% Train Validation Set repeated 100 times			689 Ev	Multi-scale PCA (Wavelet)	Empirical Classification	97.3% ± 2.7% Overall Acc for N vs CAS, 98.34% Overall Acc for N vs CAS+DAS at Ev level
[86]	2011	LOOCV			585 Ev	Temporal-Spectral Dominance spectrogram	k-NN	92.4% ± 2.9% Overall Acc at Ev level
[87]	2010	LOOCV			4-7 Rec Each	MFCC	GMM	52.5% Overall Acc on validation
[88]	2010	N/A		21 Vol, 393 W Ev	393 Ev	Continuous Wavelet Transform	Man-Whitney U Test	Significance test for features
[89]	2009	LOOCV			492 Seg	Kurtosis, Renyi entropy, frequency power ratio, Mean crossing irregularity	FDA	93.5% Overall Acc at Seg level
[90]	2009	LOOCV			2807 Seg	Fourier Transform, LPC, Wavelet Transform, MFCC	VQ, GMM, ANN	94.6% Se, 91.9% Sp for GMM using MFCC at Seg level
[91]	2009	180 Ev	-	180 Ev	360 Ev	averaged power spectrum	MLP, GAL, ISNN	Overall Acc of 98% for ISNN at Ev level
[92]	2009	75%-25% train-test split repeated 200 times			362 Ev	Lacunarity	Discriminant Analysis	99.75% maximum mean Acc at Seg level
[93]	2009	LOOCV			1544 Ev	MFCC	HMM	93.2% Se, 64.8% Sp at Ev level
[94]	2009	40 Ev, 20 W, 20 N	-	28 Rec, 112 Ev, 40 W, 72 N	152 Ev	Amplitude and Frequency of largest edge of pre-processed spectrogarm	MLP	86.1% Se, 82.5% Sp on test set at Ev level
[95]	2009	N/A		17 Rec	17 Rec	Entropy-based features	Threshold	84.4% Se, 80% Sp at Rec level
[96]	2008	40 Vol	LOOCV	25 Vol	65 Vol	AR Coefficients	k-NN, Minimum Distance-based	92% Se, 100% Sp using k-NN on test set at Rec level
[97]	2008	N/A		40 Ev	40 Ev	Peak selection based on time duration	Threshold	84% Se, 86% Sp at Ev level
[98]	2008	N/A		186 Ev	186 Ev	Distortion in Histogram of Sample Entropy	Threshold	97.9% Acc Expiration, 85.3% Acc Inspiration at Ev level
[99]	2007	N/M			870 Ev	MFCC	GMM	Acc 94.9% at Seg level
[100]	2007	N/A		18 Rec	182 C Ev	Fractal Dimension	Threshold	92.9% Se, 94.4% PPV at Ev level, 93.9% best Acc for classification
[101]	2007	3 Vol, 85 W Ev	-	10 Vol, 337 W Ev	422 W Ev	Peak selection based on local maxima, coexistence, continuity, grouping	Threshold	Se 95.5 ± 4.8%, Sp 93.7 ± 9.3% at Ev level on test set
[102]	2005	50%-50% train-test Seg from same Ev split			57 Vol	AR parameters and Cepstral Coefficients	MLP	10-20% average misclassification error on test set at Ev level for cepstral features
[103]	2005	N/A		16 Vol	16 Vol	spectrogram image	Edge Detection	Se and Sp above 89%
[104]	2005	912 Seg	114 Seg	114 Seg	1140 Seg	multi-variate AR model	BPNN	80.7% Se, 84.21% Sp at Seg level on validation set
[105]	2005	160 Ev, 80 CC, 80 FC	-	231 Ev, 158 CC, 73 FC	391 Ev	wavelet network	Discriminant Function	84% and 70% Acc for FC and CC respectively on test set at Ev level
[106]	2004	N/A		31 Vol	31 Vol	energy	Threshold	100% Se and Sp for a high airflow and 71% Se, 88.2% Sp for low airflow, at Ev level
[107]	2000	1253 Ev, 509 Ab, 744 N	repeated 5 times	1195 Ev, 530 Ab, 665 N	2448 Ev	averaged power spectrum	BPNN	Best Se 59%, 81% Sp for recorded sound and Se 87%, 95% Sp for CD data at Ev level for Ab vs N respiratory sound classification
[108]	1997	N/A		2 Rec	2 Rec	Matched wavelet	Threshold	Detection Acc of 99.8% and classification Acc of almost 100% at Seg level
[109]	1997	LOOCV			69 Vol	AR model, crackle parameters	k-NN, multinomial, voting	Overall Acc of 71.07% at Rec level to classify pathology
[110]	1996	50%-50% training-test split			13 Vol	Wavelet packet decomposition	LVQ (ANN Variant)	59% Se, 24% PPV for FC, 19% Se, 6% PPV for CC, and 58% Se, 18% PPV for W at Seg level
[111]	1995	242 Seg, 128 W, 114 N	-	2 test set: 233 Seg, 107 W, 126 N, and 235 Seg, 140 W, 95 N	710 Seg	Power spectrum	BPNN, RBF, SOM, LVQ	Overall Acc of 93% and 96% on the two sets by using LVQ at Seg level
[112]	1992	N/A		9 Vol	9 Vol	Energy envelope, Crackle characteristics	Threshold, Hierarchical clustering	100% Acc on classifying FC vs CC at Ev level
[113]	1984	42 Ev, 6 for each types	-	105 Ev, 10-15 for each types	147 Ev	LPC	Clustering (Minimum Distance)	Overall Acc of 95.24% at Ev level

Rec: Recording, Ev: Event, Seg: Segment

W: Wheeze, FC: Fine Crackle, CC: Coarse Crackle, N: Normal, Ab: Abnormal, Vol: Volunteer

CV: Cross-Validation, Se; Sensitivity, Sp: Specificity, PPV: Positive Predictive Value, Acc: Accuracy

N/A: Not Applicable, N/M: Not Mentioned

**Table 6:** Accuracy percentage measure from literature.

	WSD (%)	WED (%)	CSD (%)	WSC (%)	WEC (%)	CEC (%)
	93.8 [40] 97.9 [54] 82.1 [58] 93.25 [90] 71.2 [110]	100 [73] 85.85 [74] 85 [97] 94.6 [101] 100 [106] 79.6 [106]	83.5 [42] 84.5 [84] 99.75 [92] 99.8 [108] 62.27 [110]	93 [61] 90.5 [81] 93.5 [89] 94.9 [99] 93 [111] 96 [111]	95.15 [41] 98 [46] 95.3 [55] 75.78 [59] 98.39 [63] 97.7 [65] 98.8 [65] 100 [69] 84.75 [83] 92.4 [86] 97.5 [91] 84.3 [94] 97.9 [98] 85.3 [98]	95 [46] 98.15 [55] 89 [62] 97.5 [91]
accuracy range	71.2–97.9	79.6–100	62.27–99.8	90.5–96	75.78–100	89–98.15

### Objectives

The objective of this systematic review is to provide a summary of the existing literature on algorithms for the detection or classification of adventitious respiratory sounds. The review is organised as follows: A summary of normal and adventitious sound characteristics is provided initially. Types of analysis performed are discussed, including the adventitious sound types analysed, approach of each analysis technique, and the level at which the analyses were performed. Instrumentation and data collection methods are also provided, including sensor type, number, and position, as well as the amount of data obtained. Several works obtained data for analysis from online repositories and book audio CD companions. These databases were listed as well. A summary of data management, features, and detection or classification methods is also presented, including the performance reported in each work. Overall, a total of 77 articles are considered. This systematic review provides a complete summary of methods used in the existing literature to give a baseline for future works.

## Methods

The systematic review was performed following the recommendations of the Preferred Reporting Items for Systematic Reviews and meta-Analysis (PRISMA) statement [7]. The PRISMA checklist is provided in S1 File.

### Data sources and study selection

Studies included in this review are peer-reviewed articles written in English published between 1938 and 2016. The types of study are automatic detection or classification of adventitious sounds based on sound signal processing. No age limitation was considered as an eligibility criterion. Most data in the literature was taken from both healthy volunteers and patients with pulmonary diseases. The outcomes of the studies considered were reported as a performance measure of the automatic systems developed. The types of performance measures reported depend on the approach of each study.

The references for this review were searched using the SCOPUS (1938-2016) and IEEExplore (1984-2016) databases. Additional articles were obtained from the bibliographies of articles found. The date of the last search was 1*^st^* November 2016. Electronic search terms for these databases included adventitious sound detection, adventitious sound classification, abnormal respiratory sound detection, abnormal respiratory sound classification, wheeze detection, wheeze classification, crackle detection, crackle classification, rhonchi detection, rhonchi classification, stridor detection, stridor classification, pleural rub detection, pleural rub classification, squawk detection, and squawk classification. Articles which focused on adventitious sounds detection or classification based on breath sound with performance reported were identified from the search results. Screening was done by selecting articles based on the title and abstract. Further selection was performed on screened articles based on the eligibility criteria.

To ascertain the validity of the review, only peer-reviewed articles that provided sufficient information to approximately reproduce the results achieved were considered. Issues related to data collection and management, which may introduce bias within each study, were identified and reviewed. Thorough information on types of instrumentation or repository used, total number of data, and how the data were used are reported in the review.

### Data extraction and synthesis

Data extraction was performed by the investigators on eligible articles. A data extraction form was created to obtain important information from these articles. Extracted data were summarised into tables and further described in Section Results. Investigators extracted data about the adventitious sound type analysed, approach and level of analysis, instrumentation, location of sensor, amount of data obtained and used, data management, features, methods, and performance achieved for each study. The principal summary measure which will be used in this systematic review is the reviewed algorithm’s range of accuracy achieved for specific tasks.

A summary of normal and adventitious respiratory sounds and their characteristics is given prior to the article’s review. This summary aims to provide insight into the sounds that need to be detected or classified. Limitations of conventional auscultation are discussed next. A short description of the available commercial devices for automatic respiratory sound analysis is provided. Studies on different adventitious sound types and analysis types are identified and summarised. The different instrumentation used to collect data is also identified for each reference. The methods of analysis are discussed in separate sections. These are based on the techniques used to perform the detection or classification of adventitious sounds. The performance reported in the literature is transformed to overall accuracy where possible, for data synthesis. Balanced accuracy was used when sensitivity and specificity measures were reported instead of the overall accuracy.

## Results

This section provides the results of the systematic review performed. The section is organised as follows: A summary of normal and abnormal breath sounds is first given. This is followed by an outline of the limitations of conventional auscultation to underline the need for automated detection or classification of adventitious sounds. Commercial devices related to respiratory sound analysis are also discussed in this section. The results of the systematic review are subsequently presented. These include explanations of the type of analysis, instrumentation, and methods.

### Review of normal and abnormal respiratory sounds

Respiratory sounds are sounds generated by the respiratory system. These can usually be heard by performing auscultation. Auscultation is generally carried out to check physical health, and it involves listening to both, cardiac and respiratory sounds. Respiratory sounds heard from auscultation can be normal or abnormal. Finding abnormal respiratory sounds and differentiating them from normal sounds is important as abnormal sounds are characteristic of several serious diseases, such as asthma, COPD, and pneumonia.

#### Normal respiratory sounds

Normal respiratory sounds can be categorised based on the location where they are heard or generated. Depending on the auscultation location, different types of respiratory sounds have distinct characteristics such as duration, pitch, and sound quality. Normal respiratory sounds and their characteristics are briefly discussed below. A summary is also presented in Table 1.

- Vesicular Sounds
  Normal vesicular sounds are soft, non-musical, and can be heard on auscultation performed over most of the lung fields. Vesicular breath sounds are audible during the whole inspiration phase. However, due to the passive nature, as well as the origin, of the sounds, they can only be heard in the early expiration phase [8]. Hence vesicular sounds are longer during inspiration than during expiration. The pitch as well as the intensity are also higher in the inspiration phase compared to expiration. And while there is normally no pause between inspiration and expiration sounds in one cycle, different breath cycles are separated with a pause [8].
  Vesicular sounds have a low pitch and very limited frequency range, usually with a drop in energy after around 100—200 Hz [9]. This is due to the chest wall acting like a low-pass filter on the sounds generated. The intensity of the vesicular sounds also varies depending on the part of the chest that auscultation is performed on [8].
- Bronchial Sounds
  Normal bronchial sounds are heard over the large airways on the chest, specifically near the second and third intercostal space. Bronchial sounds are more hollow and high-pitched compared to vesicular sounds [8]. Bronchial sounds are audible during both, inspiratory and expiratory phases [10]. In contrast with vesicular sounds, due to the sounds being originated in larger airways, the expiratory phase sounds are normally audible for longer than the inspiratory phase ones. The intensity of expiration phase sounds is also higher, compared to the intensity in the inspiration phase. Unlike in vesicular sounds, there is a short pause in-between each cycle of breathing.
  Bronchial sounds contain more energy at a higher frequency bandwidth than vesicular sounds [8]. The sounds heard are usually high-pitched, loud, and tubular.
- Broncho-vesicular Sounds
  Broncho-vesicular sounds are normally heard on the posterior chest between the scapulae, as well as in the centre part of the anterior chest. The quality of the sound is between bronchial and vesicular sounds. They are softer than bronchial sounds but still mimic tubular sounds. The inspiratory and expiratory phases can be heard as having similar durations [11].
- Tracheal Sounds
  Tracheal sounds are harsh, loud, and usually have high pitch [8]. The sounds are normally heard when auscultation is performed over the trachea, specifically on the suprasternal notch. The sounds heard are usually hollow and tubular as they are generated by turbulent airflow passing through the pharynx and glottis [10]. The gap between inspiratory and expiratory phases in tracheal sounds is distinct, with both phases having a similar duration.
  The energy distribution in frequency is more spread when compared to the other normal sounds, with a much energy in the higher frequency components. The frequency range of normal tracheal sounds can reach up to 5,000 Hz with an energy drop usually occurring from 800 Hz [12]. The sounds heard over the trachea have a high intensity and can give more information as they are not filtered by the chest wall.
- Mouth Sounds
  Breath sounds heard from the mouth are produced by central airways, and caused by turbulent airflow below the glottis. Breath sounds from the mouth have a wide frequency range of 200 to 2,000 Hz [13]. The energy distribution is similar to that of white noise. For a healthy person, breath sounds from the mouth should be silent.
  The comparison and summary of the types and characteristics of normal respiratory sounds can be seen in Table 1.

Different locations for auscultation provide different sound characteristics, even for normal breath sounds. This may cause automatic analysis of lung sounds to be more complex when signals are obtained from multiple locations.

#### Abnormal respiratory sounds

Abnormal breath sounds include the absence or reduced intensity of sounds while breathing, normal breath sounds heard in abnormal areas, as well as adventitious sounds. Adventitious sounds refer to sounds superimposed on normal breath sounds. These can be characterised based on the underlying conditions and hence be very useful in helping diagnosis. Adventitious sounds can be classified into two categories, continuous and discontinuous, based on their duration.

- Continuous Adventitious Sounds
  Continuous Adventitious Sounds (CAS) are abnormal sounds superimposed on normal breath sounds with durations of more than 250 ms [14]. Based on the pitch, CAS can be further categorised as high-pitched (Wheeze, Stridor, and Gasp) or low-pitched (Rhonchi and Squawk). Based on the associated condition and cause of the adventitious sounds, different types of CAS can also be separated.
  - Wheeze and Rhonchi
    Wheeze and rhonchi are both continuous adventitious sounds which can be heard during inspiration, mostly at expiration, or during both phases [10]. Wheeze is a high-pitched CAS while rhonchi are low-pitched. Wheeze sounds are caused by the airway narrowing which then causes an airflow limitation [15] while rhonchi are related to the thickening of mucus in the larger airways [16]. According to [17], although wheeze and rhonchi belong to CAS, they do not necessarily have durations of more than 250 ms. Some have reported that wheeze and rhonchi can have minimum durations of around 80 to 100 ms.
    Wheeze and rhonchi both present as sinusoid-like signals, with frequency ranges between 100-1,000 Hz. Wheeze is defined as a high-pitched continuous sound with dominant frequency of a minimum of 400 Hz, while rhonchi is a low-pitched continuous sound with dominant frequency of a maximum of 200 Hz [14]. Both wheeze and rhonchi are musical sounds, usually with up to three harmonic frequencies [18].
    Diseases associated with wheeze are asthma and COPD. If the wheeze is localised, it may be caused by a foreign body blocking the airway, like a tumour [10]. Rhonchi is associated with COPD and Bronchitis due to the secretions in the bronchial tree [10].
  - Stridor
    Stridor is a type of CAS with a sibilant and musical quality, similar to wheeze. Stridor can mostly be heard on the inspiration phase although, on some occasions, it can be heard on expiration or even in both phases [10]. Different from wheeze, stridor sound is generated by turbulent airflow in the larynx or bronchial tree, and is related to an upper airway obstruction. This is why stridor can be heard more clearly on the trachea, while wheezing can also be heard clearly by chest auscultation [19]. Stridor sounds are characterised by a high pitch of more than 500 Hz [10]. They are also normally harsher and louder than wheeze sounds. As a type of CAS, stridor sounds have a duration of more than 250 ms.
    The differential diagnosis for stridor are epiglottitis, croup, and laryngeal oedema. All of these conditions are related to upper airway obstructions. Stridor sounds can also be heard when there is a foreign body such as a tumour in the upper airway tract.
  - Gasp
    Inspiratory gasps can be heard usually after a bout of coughing when a patient finally tries to inhale. The whoop sound of an inspiratory gasp is caused by fast moving air through the respiratory tract. Whoop sounds typically have a high pitch and long duration, which makes inspiratory gasps belong to CAS. The whooping sound is a pathognomonic symptom of whooping cough (pertussis) [20]. This is the only disease associated with a whooping sound inspiratory gasp.
  - Squawk
    Squawks are adventitious sounds that can be heard during the inspiratory phase. The sound is a mix of both musical and non-musical. Squawk is also called short wheeze as the sound’s characteristics are similar to a low-pitched wheeze but with a shorter duration [8]. The pitch of squawk is normally between 200–300 Hz [10]. The sounds are generated by oscillation at the peripheral airways [21]. Squawk can usually be heard in a patient with hypersensitivity pneumonia, although they have been reported in patients with common pneumonia several times [22].
- Discontinuous Adventitious Sounds
  Discontinuous Adventitious Sounds (DAS) are abnormal sounds superimposed on normal breath sounds with a short duration of less than 25 ms [14]. DAS can be further classified based on the source from where the sounds are generated.
  - Fine Crackle
    Fine crackle sounds are caused by explosive openings of the small airways. The sound is high-pitched (around 650 Hz) and has a short duration (around 5 ms) [23]. Crackle sounds are explosive and non-musical [8, 24]. Fine crackles are audible only at the late stages of inspiratory phases. Fine crackle sounds are usually associated with pneumonia, congestive heart failure, and lung fibrosis.
  - Coarse Crackle
    Coarse crackle sounds are generated by air bubbles in large bronchi. The sounds can be heard mostly during the early stages of inspiration, but are also audible at the expiratory stage. Coarse crackles have a low pitch, around 350 Hz, with a sound duration of around 15 ms [23]. Coarse crackle sounds can be heard on patients with chronic bronchitis, bronchiectasis, as well as COPD.
  - Pleural Rub
    Pleural rub are non-musical rhythmic sounds, which are categorised as DAS as the duration of each rub is around 15 ms [10]. Pleural rub sounds are caused by the rubbing of pleural membranes when breathing. The sound generated by the friction can be heard on both phases (biphasic), inspiration and expiration. Pleural rub sounds have a low pitch, normally below 350 Hz [10]. They are usually caused by inflammation of the pleural membrane [8]. Pleural tumour can also cause them [10].

Table 2 provides a summary and comparison of the different adventitious sounds. From Table 2, it can be seen how developing a classification tool for adventitious sounds is a challenging task, since there is a significant overlap between the characteristics of different sounds. In addition, typical characteristics may not be general and representative for different patients.

#### Auscultation

Auscultation is the medical term referring to the use of a stethoscope or other tools to listen to the sounds generated from inside the body. It is used to help diagnose a vast number of conditions. Normally, auscultation is performed to listen to lung, cardiac, abdomen, and blood vessel sounds. Most of the time, auscultation is performed on the anterior and posterior chest [25].

The stethoscope used for auscultation usually consists of two parts, a diaphragm and a bell. The diaphragm is used to listen to high-pitched sounds while the bell is for low-pitched sounds. Auscultation is recommended to be performed in a quiet environment to enable the expert to listen to the sounds clearly [8].

#### Drawbacks and Limitations of Conventional Auscultation

The first limitation of conventional auscultation is that it cannot be performed frequently and thus cannot provide continuous monitoring. Auscultation needs to be performed by an expert, especially when trying to detect and determine abnormal sounds. This is very limiting, for example, in the case of asthma, because symptoms such as wheezes most often occur during the night. The requirements of performing auscultation in a quiet environment, and ideally with the patient in a still position, are also very restrictive.

The number of people capable of performing auscultation is also limited. An expert on auscultation needs to have lot of experience in order to be able to determine the types of sounds heard and decide on how this information can help in diagnosis or monitoring. Symptoms might be missed and their severity underestimated by both patients and physicians [26], resulting in proper care not being given.

Limitations of the human auditory system are also a drawback in conventional auscultation. A study in [27], advocates that conventional auscultation should not be used as a reference in research on automatic lung sound analysis. The intensity of respiratory sounds can mask the adventitious sounds, resulting in only normal sounds being heard. The varying amplitude of adventitious sounds may also cause the human ear to miss some cases where the intensity is too low to be detected.

These limitations and drawbacks hinder the effectiveness of conventional auscultation as a mean of monitoring and managing symptoms. Automated lung sound analysis, specifically automatic detection and classification of adventitious sounds, could potentially overcome these limitations.

#### Available Automated Lung Sound Analysis Devices

Automatic lung sound analysis, aiming to overcome the limitations mentioned above, has been the recent focus of a significant amount of research, and some commercial systems for very specific applications are already in the market [25]. These include the Wheezometer [28], Wholter [29], VRI [30], LSA-2000 [31], LEOSound [32], Multichannel STG [33], STG for PC [34], and Handheld STG [35].

Wheezometer and WHolter were developed by Karmelsonix (now Respiri). Wheezometer is used to measure the wheeze percentage and uses one sensor placed over the trachea. WHolter has a similar sensor and algorithm to Pulmotrack [36], but is intended for home monitoring use. The data recorded by WHolter is uploaded to a computer to be analysed. Vibration Response Imaging (VRI) developed by Deep Breeze uses 34 or 40 sensors placed on the posterior chest. The device is capable of detecting lung vibration energy and visualises it in a grayscale image. LSA-2000, by Kenzmedico uses up to 4 sensors attached over the chest to identify interstitial pneumonia. LEOSound developed by Heinen and Lowerstein uses 3 sensors capable of storing data for wheeze and cough detection. Multichannel STG uses 14 sensors placed on multiple locations on the posterior chest, trachea, and an over the heart sensor. The device is capable of counting crackles, rhonchi and wheezes. Smaller versions of STG use an electronic stethoscope coupled with either a PC (STG for PC) or a handheld device (Handheld STG).

Automated lung sound analysis devices should be easy to use, portable, and require as small a number of sensors as is possible [25], The use of multiple sensors and bulky devices is not suitable and cost-effective for home monitoring purposes. All the devices listed above are typically large and complex, with the exception of the Wheezometer, but this can only provide spot-checks, not continuous monitoring. WHolter has portability but works as data logger with a separate analysis device. While STG for PC and Handheld STG use an electronic stethoscope that is also not suitable for continuous monitoring. Thus, portable or wearable non-intrusive devices that can be used to monitor lung sounds without the help of experts are still needed.

Other than the devices mentioned above, the development of algorithms to detect or classify lung sounds has been the focus of a lot of research works. These works developed detection or classification methods by extracting certain features from the sounds. The detection and classification methods used vary from empirically determined to the use of machine learning. A systematic review of automatic detection or classification of adventitious sounds is presented in next subsection.

### Review of algorithms for automatic adventitious respiratory sound analysis

This section reviews published studies on the detection or classification of adventitious respiratory sounds. The review is organised as follows. The types of sound being investigated will be discussed first. This is followed by a discussion of the level at which the analysis is performed. The sensor types, number, and placement is reviewed next. Available online databases with recordings of adventitious sounds are presented. The methodology of analysis is reviewed last, including the use of the data, validation, features, and the classification or detection methods used.

#### Study selection

A total of 77 full-articles were included in this systematic review. Database search on SCOPUS and IEEExplore, as well as citation tracking identified a total of 1519 records. Removal of duplicates and non-accessible full-text articles left 1446 articles. Out of these, 1297 articles were excluded based on title and abstract screening. From the screening, 149 full-text articles were then assessed for eligibility, and 72 studies were excluded. This study selection resulted in a total of 77 eligible full-articles which were all included in the review. The flow diagram for this study selection can be seen in Fig 1.

Characteristics of studies included in this systematic review are given in Tables 3 and 4. The characteristics summarised for each work are: type of sound analysis, approach and level of analysis, instrumentation or database used to obtain data, and amount of data used in the analysis.

#### Types of sounds analysed

Although all eligible articles included in this review targeted adventitious sounds, different works had different specific aims. Hence, some of the works investigated one type of adventitious sound and compared it with normal breath sounds- this can be performed as a detection or classification scheme. Others reported the classification of several types of adventitious sounds. There were also works that performed classification on the cause of adventitious sounds generation.

Examples of the analysis performed in the published papers included: wheeze detection, wheeze classification against normal breath sounds, classification of monophonic and polyphonic wheeze, crackle detection in a recording, and classification of crackle and normal breath sounds. Other than wheeze and crackle analysis, adventitious sounds analysis was performed in combination in different works. Generally, the analysis was on classification tasks, such as: wheeze and rhonchi classification, classification of wheeze and crackle, wheeze and stridor classification, and other combinations. Another example was classification between sounds caused by airway obstruction or parenchymal. 55 (71.43%) of the studies focused on wheeze, 40 (51.95%) on crackle, 9 (11.69%) on stridor, 9 (11.69%) on rhonchi, and 18 (23.38%) on other sounds such as pleural rub, squawk, as well as the pathology. A summary of the types of sounds analysed in each article can be seen in Table 3.

#### Level of analysis

There are three different levels of adventitious sound analysis that can be performed. Several studies performed detection and classification of adventitious sounds at a segment level. For detection at the segment level, features are usually extracted on segments generated by signal windowing. Classification may also be performed at the segment level. Random segments from both, adventitious and normal sounds, are obtained and used to perform this classification. Different from classification at the segment level, classification at the event level is usually done after obtaining manually isolated events of adventitious sounds and normal breath sounds. At the recording level, the task performed is usually the detection of events.

Different levels of analysis result in different performance measures. At the segment level, one possible performance measure is to regard each segment as either true positive, true negative, false positive, or false negative. Another approach is to combine the detected segments, for example by taking a few consecutive detected segments as a positive event or by taking the mean values of extracted features. For the reported works using the event level (usually a classification task), the performance is measured from individually isolated events. Detection tasks performed at the recording level measure the performance at the event level. As for classifications performed at the recording level, the analysed recording will either be classified as containing abnormal sounds or as a normal recording. More detail on how each work in the literature performed analysis and measured the performance can be seen in Table 3.

#### Sensor and its placement

Most research works on adventitious sound analysis used data recorded from patients in hospital. The most common sensors being used for data collection were microphones. The types of microphone mentioned were the SP0410HR5H-PB [114], KEC-2738 [115], TSD108 [116], Panasonic WM-61 [117], SONY ECM-44 BPT, and SONY ECM-77B [118]. Several articles also used microphones but without mentioning the type specifically. Electronic stethoscopes were also used by several researchers. These include the ThinkLab Rhythm:ds32a Digital Stethoscope [119], WelchAllyn Meditron Electronic Stethoscope [120], and Littmann 3M Electronic Stethoscope Model 4000 [121], and 3200 [122]. One paper used an accelerometer BU-3173 [123] as a sensor. Other than the sensors above, several studies stated the use of either a microphone or stethoscope without specifically mentioning the type. In total, there were 31 studies that used microphones and 21 studies that used electronic stethoscope.

Conventional auscultation is usually performed on the anterior and posterior chest in order to obtain vesicular breath sounds. For the development of algorithms for the detection or classification of adventitious sounds, several studies used the trachea, specifically the suprasternal notch, as the location for the sensor. Mouth breath sounds were also used in one of the papers to detect wheezes.

The number of sensors used to perform the analysis varies from only one sensor up to a set of 14. In some papers, although only one sensor was used, the sensor is not kept in a fixed position but it is used to detect sounds from multiple locations, similar to performing conventional auscultation. This was generally the case when the analysis was performed using a digital stethoscope for data collection. A summary of the sensors used in each work can be seen in Table 4.

#### Databases

Several works used available databases as a source for analysis instead of collecting their own data. The databases used are from online repositories and from audio CD companion books. The online repositories available were from R.A.L.E [124], East Tennessee State University repository [125], Littmann repository [126], and from SoundCloud [127]. The audio CDs companion used were from books such as Understanding Lung Sounds 2*^nd^* Edition [128], Understanding Lung Sounds 3*^rd^* Edition [129], Auscultation Skills: Breath and Heart Sounds [130], Fundamentals of Lung and Heart Sounds [131], Understanding Heart Sounds and Murmurs [132], Heart and Lung Sounds Reference Library [133], Secrets Heart & Lung Sounds Workshops [134], Lung Sounds: An Introduction to the Interpretation of the Auscultatory Finding [135], and The Chest: Its Signs and Sounds [136].

Breath sounds from online or book databases were taken from multiple locations, such as the chest, neck, and mouth. The sensor used for the data collection varied and included an electret microphone and accelerometer in [124], and the Littmann Digital Stethoscope in Littman repository [126].

#### Method of analysis and performance

Algorithms developed to detect or classify adventitious sounds usually involve two steps. The first step is to extract the relevant features that will be used as detection or classification variables. The second step is to use detection or classification techniques on the data, based on the features extracted. In developing a detection or classification algorithm, especially if machine learning techniques are used, it is important to take note of how the data is used to train, test, and validate the algorithm. In this section, the literature published will be discussed. The following aspects were reviewed: features extracted; classifier or detection techniques used; how the training, testing, and validation was performed; as well as the performance achieved. The section is organised based on the classifier or detection techniques used. These are empirical rule-based (such as with thresholding or peak selection), Support Vector Machine (SVM), Artificial Neural Network (ANN) variant, and other techniques such as clustering and statistical models. Table 5 is provided to summarise the review.

#### Empirical Rule Based Methods

A study by [62] performed crackle classification. The data used included 50 crackle events and 50 normal breath sounds. The sounds were recorded using a Littmann 3M 4000 Electronic Stethoscope at multiple positions on the chest. The classification performed was based on the mathematical morphology of a crackle event in the spectrogram. The classification achieved 86% sensitivity with a specificity of 92%.

Wheeze classification was performed in [95]. The data used for the study was obtained from [129]. A total of 17 recordings, with 7 normal and 10 containing wheezes were used. The classification performed was to determine whether a recording was normal or contained wheezes. The feature used was extracted based on the entropy of each frame of the segmented recording. The feature set was the ratio and difference of the maximum and minimum entropy of the segments of a recording. Based on an empirical threshold, the classification was performed. The study achieved 84.4% sensitivity and 80% specificity.

A empirical threshold was also used as a classifier by [50] to perform multi-class classifications between wheeze, stridor, crackle, and normal events. This study was a continuation of [95] above. The data used for this study was obtained from both hospital and the Soundcloud online repository with the search term ‘lung sounds’. A total of 45 recordings were used, containing several cycles of respiration each. Similar to the algorithm in [95], entropy was extracted from the segmented recording. For the multi-class classification, two entropy-based features were extracted instead of just one as in the previous study. The entropy-based features were the difference and ratio of maximum and minimum entropy of a segment in a recording. Similar to [95], the performance was measured by classifying a whole recording using the extracted features. The performance reported was 99% for stridor, 70% for wheeze, 87% for crackle, and 99% for normal sounds.

A finding from [63] claimed that the delay coordinate can be used as a feature to perform a classification between wheeze events and normal breath sounds, achieving 98.39% overall accuracy. The underlying reason was that the wheeze sound signal is a sinusoid while a normal breath sound is noise-like. A threshold can be found to perform the classification based on the persistent homology of delay embeddings. Another study from the same group [73] previously focused on wheeze sound detection in a recording. The data used contained 6 wheeze events in a recording which could all be detected using an energy threshold classifier on certain frequency bands and wavelet packet decomposition.

Wheeze detection was also studied by [77], with signals obtained using a stethoscope that was built using a microphone inside a chamber. The sounds were recorded from the neck. A total of 59 recordings, 25 with wheezes and 34 normal, from 8 young children were used for analysis. The feature used was the correlation coefficient, while the classifier was an empirically determined threshold. The features were extracted from each segment of a recording. Several consecutive high correlation coefficients were regarded as a wheeze event. Finally, each recording was classified as containing wheeze or being normal by using a threshold, calculated as the ratio between wheeze duration and normal respiratory duration. The performance achieved was 88% sensitivity with 94% specificity.

The study in [74] also focused on wheeze detection. The wheeze sounds were recorded using a single digital stethoscope from multiple positions. In total, 40 recordings were used for the study. The features were obtained from time-frequency analysis, with a rule-based decision making, such as finding and selecting peaks based on energy threshold, derived from the algorithm developed by [101]. The study achieved 72.5% specificity with a sensitivity of 99.2%.

Classification of CAS and DAS against normal breath sounds was carried out by [80]. 47 recordings from an online repository [124] were used. These contained 10 normal, 20 CAS, and 17 DAS recordings. There were two features analysed in this study. The first feature was a similarity measure of segments in the recording using mutual information. The second feature was a weighted cepstral feature. The study claimed a high accuracy of classification by using a threshold classifier using the first feature, while a separability index of 1 was found using the second set of features for both CAS and DAS classification.

Wheeze segment classification was performed in [81], also using a threshold-based classifier. A total of 180 segments were analysed. These contained 82 wheeze segments and 98 normal segments. The feature used in this study was the fractional Hilbert transform. The overall accuracy achieved was 90.5%. The same research group performed crackle detection also using the fractional Hilbert transform as a feature in [82]. The correlation coefficient was used as additional feature to detect crackle. The performance achieved was a sensitivity of 94.28% and Positive Predictive Value (PPV) of 97.05%, at the event level, on 10 short recordings with 33 crackle events.

Crackle detection was also performed in [56] by using thresholding on fractal dimension and the CORSA [143] criterion of crackle. A total of 24 recordings were used for the analysis, obtained using a stethoscope. The performance reported was an average sensitivity of 89 ± 10% and PPV of 95 ± 11%, at the event level, for different recordings.

A study in [84] also performed crackle detection using a threshold-based classifier. The feature used in this study was the abnormality level. A total of 433 segments were used in the analysis with no further detail given. The performance reported was 84.5% accuracy.

Wheeze detection was performed in [83] using the Linear Predictive Coding (LPC) prediction error ratio as a feature. A total of 26 recordings were used for analysis, with 13 of them containing wheeze sounds. By using a threshold classifier on the prediction error, 70.9% sensitivity and 98.6% specificity at the event level was achieved.

The work in [97] used peak selection based on time duration to perform wheeze detection. A total of 40 events were obtained from several databases. The only currently available database is [125]. From the 40 events, 19 of them were wheezes and 21 were normal respiratory sounds. The performance reported was 84% sensitivity and 86% specificity.

Wheeze and normal respiratory event classification was performed in [98]. Signals from 14 volunteers were recorded using one SONY ECM-77B microphone. An additional 100 normal and 86 wheeze events from [129, 132] were obtained. The classification was done using distortion in histograms of sample entropy as a feature. Performance of 97.9% accuracy for expiration and 85.3% accuracy for inspiration phase, at the event level, was reported.

Threshold on fractal dimension was used to perform the detection of crackle segments in [100]. A total of 18 recordings with 182 crackle events were analysed. 92.9% sensitivity and 94.4% PPV, at the event level, detection of crackle were achieved.

The work in [106] performed wheeze detection with signals obtained from 16 asthmatic patients and 15 healthy volunteers. Data were recorded using one piezoelectric microphone placed on the neck. A threshold energy was used achieving 100% sensitivity and specificity for high airflow at the event level. Wheeze detection was also the focus of the study in [101]. Signals from 13 volunteers containing 422 wheeze events were recorded using five SONY ECM-77B microphones placed on the neck, anterior, and posterior chest. Data from 10 out of 13 volunteers were used as a test set containing 337 wheeze events. The detection was made by selecting peaks based on sets of rules. Sensitivity of 95.5 ± 4.8% and specificity of 93.7 ± 9.3%, at the event level on the test set, was achieved.

The study in [108] studied both crackle segment detection and classification using signals obtained from the ACCP teaching tape. The feature used for detection was the correlation between a crackle signal in the time domain with a wavelet decomposition. The crackle segment detection achieved 99.8% accuracy. Classification between fine and coarse crackle was performed on the detected crackle segments. The article claimed that the achieved accuracy was “almost” 100%.

Prior to this, [112] also performed crackle detection and classification. A threshold on energy envelope was used to detect and isolate crackle segments. The detected crackles were further classified into fine or coarse by using crackle typical characteristics such as peak frequency and time duration. The algorithm was applied to signals from 9 patients obtained using a microphone. The study claimed to achieve 100% accuracy in classifying crackles into fine or coarse.

#### Support Vector Machine Based Methods

The work in [37] used an SVM classifier to perform wheeze detection. The signals used were obtained with a single microphone (SP0410HR5H-PB) used to record mouth breath sounds. A total of 95 recordings were collected, with 27 of them containing wheezes. 70 recordings with wheezes in 20 of them, were used to train the SVM classifier while the rest were used to test the classifier. A separate set of 39 recordings with 10 wheezes were used as an additional test set. Spectral-based features were used for the classifier. The recordings were divided into segments and the features were extracted from each frame of the segmented recordings. Using this method, 71.4% sensitivity and 88.9% specificity was achieved on the validation set at the recording level. Logistic Regression Model (LRM) classifier was also used, but the result using SVM achieved a better overall performance.

A study in [41] used five TSD108 microphones to obtain recordings from 30 volunteers to be used for CAS classification. In total, 870 inspiratory cycles, from which 485 samples containing CAS, were recorded. Four of the sensors were placed on the back while one sensor was put on neck. From the 870 cycles, 1494 segments were obtained with 633 of them containing CAS. A feature set based on instantaneous frequency was extracted and an SVM classifier was used. To obtain the optimal SVM parameters, 10-fold cross-validation (CV) was used, using 559 cycles out of the 870 recorded. The SVM model was then developed using 100 iterations of 65%-35% of random data, split out of the 1494 segments. If at least one segment in a cycle was classified as CAS, the whole cycle would be classified as CAS. The best performance obtained was a sensitivity of 94.2% and a specificity of 96.1% at the cycle level.

The study in [38] used SVM to perform classification of recordings using a denoising autoencoder as feature set. The data for the study was recorded using a stethoscope on the neck, anterior, and posterior chest. A total of 227 recordings were obtained, 171 normal, 33 containing wheeze, 19 containing crackle, and 4 containing both wheeze and crackle. The performance achieved was 90% sensitivity with 64% specificity for wheeze and 90% sensitivity with 44% specificity for crackle at the recording level.

The same research group built a custom stethoscope and algorithm in [47] to perform wheeze detection. The detection scheme used consisted of processing the spectrogram of sound recordings to select potential wheezes by using the energy threshold, and performing the classification on selected potential wheezes to obtain the final classification result for the recording classification. The performance achieved was 86% accuracy at the recording level, by taking into account the expected number of false positives.

Classification of normal, wheeze, and crackle events was performed in [46] using k-Nearest Neighbour (k-NN) and SVM. A total of 600 events, with 200 normal, 200 wheezes, and 200 crackles were obtained using fourteen SONY ECM-44 BPT microphones. Leave-one-out cross-validation (LOOCV) was used with energy and wavelet coefficients as features. The best performance was achieved by using SVM. This was 95.17% average accuracy at the event level.

Differentiating between monophonic and polyphonic wheezes was performed by [59]. The recording of the wheezes was carried out using fourteen microphones (SONY ECM-44 BPT) positioned on multiple locations on the chest. A total of 7 recordings containing 121 monophonic and 110 polyphonic wheezes were used for analysis. A SVM was used as the classifier with quartile frequency ratio and mean crossing irregularity as features. The SVM performance reported was 69.29% accuracy. k-NN and Naive Bayes (NB) classifiers were also used. The best overall accuracy reported was 75.78%, achieved using k-NN.

Wheeze detection using Mel Frequency Cepstral Coefficients (MFCC), kurtosis, and entropy as features was developed in [53]. 45 recordings for the analysis were obtained using an accelerometer (BU-3173). Two parallel SVMs were used as classifiers with a final decision made using the product of the outputs of both. 21 recordings were used for training while the rest were used to test the model. 20%-80% data split was used for validation (repeated 20 times). The performance was reported as a reliability measure, which was defined as the true positive rate times the true negative rate. The reliability reported was 97.68%.

Another wheeze and normal sound classifier was developed in [61]. The detection was performed at the segment level, with data obtained from online repositories [125, 126] and their own recordings. The data used contained 130 wheeze segments and 130 normal segments. A SVM was also used as a classifier, with audio spectral envelope variation and a tonality index as features. A 10-fold CV was performed, with accuracy reported of 93%.

A C-weighted SVM was used in [58] to perform wheeze detection. Data for the study was obtained from [124], which included 26 recordings. A total of 1188 segments were annotated; 290 of them were wheeze segments. Leave-two-out cross-validation (LTOCV) was used in such a way that one of each normal and wheeze segments were used as a test set. MFCC, wavelet packet transform, and fourier transform features were used and compared. The performance achieved was 81.5 ± 10% sensitivity and 82.6 ± 7% specificity for MFCC features to detect wheeze segments.

Crackle and rhonchi classification was presented in [64]. 60 recordings were used for analysis, obtained using a WelchAllyn electronic stethoscope at multiple positions on the back. The frequency ratio, average and exchange time of instantaneous frequency, and eigenvalues were used for feature extraction. The feature set was extracted from each frame of the segmented recordings. 5-fold CV was used with a SVM as a classifier. The performance was obtained using each of the features, with one-versus-one and one-versus-all SVM classifiers. The accuracy was above 80% for all cases.

The work in [65] developed new features to perform CAS classification. The CAS analysed were wheeze, stridor, and rhonchi. Data for the study was obtained from both volunteers and databases. The volunteer’s signals were recorded using a SONY ECM-77B microphone positioned on the trachea, while the databases used were from [129, 131, 132]. From the data collection, 339 events were obtained. The data from the database contained 239 events. A feature set of size 5 was obtained after performing feature selection. The features were extracted based on instantaneous kurtosis, discriminating functions, and sample entropy. LOOCV was used with a SVM classifier achieving accuracy of 97.7% for the inspiration cycle and 98.8% for the expiration cycle.

Differently from the other works here, the study in [75] performed classification on the cause of adventitious sounds. The two classes for the classification were airway obstruction and parenchymal pathology. The data used for the study was obtained from [124] which contained 68 recordings. The recordings consisted of 17 normal, 26 with airway obstruction, and 25 with parenchymal pathology. The classification was performed with 60%-40% train-validation set repeated 25 times. MFCC were used as features with a SVM classifier, achieving an accuracy of 94.11% for classifying normal recordings, 92.31% for airway obstruction pathology, and 88% for parenchymal pathology.

A SVM classifier was also used in [76] to perform a classification between crackle and normal sounds. Signals were obtained using fourteen SONY ECM-44 BPT microphones positioned on the chest. A total of 6000 segments with 3000 of them being crackle sounds were extracted from 26 different recordings. The data were split evenly for training, test, and validation of the SVM model. Multilayer Perceptron (MLP) and k-NN methods were also used for the classification. The performance was reported separately for each classifier. The study found that the SVM was superior to the k-NN and MLP, with an overall accuracy of 97.5% and sensitivity of 97.3%.

Another work which used a SVM as classifier was [78]. The focus of this study was to perform classification between normal and abnormal breath sounds. A ThinkLab digital stethoscope was used to obtain 28 recordings for the analysis. Out of the 28 recordings, 10 of them were normal, 10 contained wheezes, and 8 had crackles. A cortical model of the recordings was extracted as a feature, and 10-fold CV was performed. The performance achieved was 89.44% for sensitivity and 80.5% for specificity.

#### Artificial Neural Network Variant Methods

A MLP was used in [102] to perform a classification of respiratory sounds from 20 healthy volunteers, 18 patients with obstructive, and 19 patients with restrictive disorder. 50%-50% train-test set was used with Auto Regressive (AR) parameters and cepstral coefficients as features. The performance achieved was 10-20% average misclassification error on the test set at the event level for the cepstral coefficient feature set. Further post-processing was performed to increase the accuracy of the classification at the recording level.

A MLP classifier was also used in [94] to perform the classification of wheeze and normal events. The data for the classification was obtained from the online repository [124], Ausculta pulmonar, and IMD 420-C review of lung sounds. A total of 28 recordings with 40 wheeze events and 72 normal events were used to test the MLP classifier. For the MLP training, 40 separate events were used with 20 of them being wheeze events. A set of features with a size of 20 were extracted. The features were obtained from the amplitude and frequency of the 10 largest edges in a pre-processed spectrogram. The spectrogram of each event was pre-processed using a Laplacian mask. The result of the MLP wheeze classifier was an 86.1% sensitivity and an 82.5% specificity.

The work in [69] also used a MLP to perform the classification of wheeze, crackle, and normal breath sounds. The data was obtained from an online repository [124]. 13 events, with 4 containing wheeze, 4 containing crackle, and 5 normal were used with a LOOCV technique. The features used were 13 MFCCs. The recordings were first windowed and each segment was classified using the MLP. The event classification was performed based on the segment classification. An event was classified as a certain class if most of its segment’s were classified as that class. The event classification achieved individual accuracy of 100% for wheeze, 75% for crackle, and 80% for normal sounds.

A MLP was also used as a classifier in [45]. The features used were 20 MFCCs. The data used for the study was obtained from an online repository [124] and from the IIT Kharagpur Institute of Pulmocare and Research Kolkata. 30 recordings containing 72 events were obtained, with 24 of them normal, 24 containing wheezes, and 24 others with crackle events. The LOOCV technique was used, achieving a 97.83% overall accuracy of classification. Other cepstral-based features were also discussed, such as: Linear Prediction Cepstral Coefficient (LPCC), Perceptual Linear Prediction Cepstral Coefficient (PLPCC), Linear Frequency Cepstral Coefficient (LFCC) and Inverted MFCC. These cepstral features were compared with wavelet-based features. The study concluded that cepstral-based features achieved better accuracy than wavelet-based ones.

The study in [55] used a Fuzzy Neural Network (FNN) to perform a classification on abnormal and normal breath sounds. The normal breath sounds in the study consisted of bronchovesicular, normal bronchial, normal bronchophony, and normal egophony. The abnormal sounds included crackles, wheezes, abnormal bronchial, stridors, bronchophony by consolidation, and egophony. The sounds were obtained from [129, 130] audio CD book companion which contains 28 recordings. The data was split into 70%-15%-15% train-test-validation set. The features were extracted from the power spectral density of each events. The power spectrum was averaged into 32 frequency ranges, such that the feature vector was of size 32. The performance on the test set was 97.8% sensitivity with 100% specificity for abnormal sounds classification.

A back propagation neural network (BPNN) was used by [107] to perform the classification of abnormal and normal respiratory sounds. Data was recorded using two LS-60 microphones placed on the anterior chest. Additional data from [129, 131] were also obtained. The best performance achieved was a sensitivity of 59% and 81% specificity for recorded sounds, and a sensitivity of 87% and 95% specificity for CD additional data at the event level for abnormal respiratory sound classification. The feature used was averaged power spectrum.

The study in [104] used BPNN to perform segment classification of crackle and non-crackle. Data was recorded using 25 microphones placed on the posterior chest of 10 healthy volunteers and 19 patients. 912 segments, of which 456 were normal and 456 were abnormal, were used to train the BPNN. 114 segments were used for validation while another separate 114 segments were used as a test set. A multi-variate AR model was used as a feature, achieving 80.7% sensitivity and 84.21% specificity, at the segment level on the validation set.

BPNN was also used by [49] to perform recording classification. The study used 58 recordings with 32 of them containing wheezes obtained using an ECM microphone. 13 wheeze and 10 normal recordings were used for training, while the rest were used to test the neural network. Before using the BPNN, potential wheeze episodes were first selected from the recordings by using the Order Truncate Average (OTA) method to preserve peaks. The peaks were further processed using a threshold to obtain potential wheezes. These potential wheezes were then classified using a BPNN. The features used were the duration, frequency range, boundary, normalised power spectra, and slope of the potential wheeze. The performance claimed by the study was high, with a sensitivity of 94.6% with 100% specificity for wheeze recording classification.

Extreme Learning Machine (ELM) was used to perform a classification between abnormal and normal sounds in [68]. The abnormal sounds that were analysed included wheeze, crackle, and squawk sounds. The data was taken using a microphone placed on the trachea. A total of 30 recordings were obtained, from which 120 cycles were annotated. A 5-fold CV technique was used for the classifier. The feature vector for the classification consisted of lacunarity, sample entropy, kurtosis, and skewness of the event power spectrum. SVM classifier was also discussed in this study. The performance for the ELM classifier was 86.30% for sensitivity and 86.90% for specificity when the whole set of features were used. When the SVM classifier was used, 86.30% for sensitivity and 85.80% for specificity was achieved, also with all features used.

The work in [72] performed an analysis of wheeze and crackle using signals from patients with tuberculosis. The recordings for the analysis were taken using 7 microphones positioned on the neck, chest, and back. Signals from 60 volunteers were obtained. An Artificial Neural Network (ANN) was used with 75% of the data for training and 25% to test the model. The classification performed was to check if a recording was from a patient with tuberculosis or a normal one. The presence of a wheeze was detected by evaluating the spectrogram while crackles were identified using wavelet-based features for the ANN. The performance obtained was a sensitivity of 80% with a specificity of 67% in detecting tuberculosis.

An ANN was used in [71] to perform event classification of respiratory sounds containing wheezes and crackles. Data was obtained from [129]. A total of 92 events with 27 normal, 31 crackles, and 34 wheezes were obtained. 60 events were used for training, 14 events were used for validation, and 18 events were used for the test set. A wavelet packet transform was used as the feature set, achieving a 98.89% best average accuracy for Symlet-10 wavelet base on the test set.

Multiple variants of ANNs were used and compared in [91]. The classification task performed was to differentiate wheeze, crackle, stridor, squawk, pleural rub, and other types of sounds using a MLP, a Grow and Learn network (GAL), and an Incremental Supervised Neural Network (ISNN). A total of 360 events from 36 recordings were obtained. An averaged power spectrum was used as a feature, achieving a best accuracy of 98% for the ISNN classifier on a test set of 180 events.

The study in [110] used a Learning Vector Quantisation (LVQ) to detect wheeze and crackle segments. The feature used was a wavelet packet decomposition. Signals recorded from the chest of four healthy volunteers and nine patients were used for the analysis. A 50%-50% train-test data split was used. This study reported a performance of 59% for sensitivity and 24% for PPV for wheeze detection. For fine crackle detection, only 19% for sensitivity and 6% for PPV was achieved while 58% for sensitivity and 18% for PPV was the reported performance for coarse crackle detection.

Wheeze segment classification using several ANN variants was performed in [111]. The ANN variants used were BPNN, Radial Basis Function (RBF), Self-organising Map (SOM), and LVQ. A total of 710 segments, with 375 containing wheezes, were used for the classification. The data was split into three sets, one training and two test sets. The training set consisted of 242 segments where 128 of them contained wheezes. The first test set consisted of 233 segments with 107 wheeze segments, while the second test set had 235 segments with 140 wheeze segments. The feature used for the neural networks was extracted from the power spectrum of the segments. Highest overall accuracy of 93% on the first and 96% on the second test sets were achieved using LVQ.

#### Gaussian Mixture Model Based Methods

A MFCC coupled with a Gaussian Mixture Model (GMM) was used in [99] to perform a classification of wheeze and normal sounds. Data for the study was taken from 30 volunteers. The instrumentation used to record respiratory sounds was an ECM microphone and a 3M Littmann Classic S.E. stethoscope placed on the neck. The study reported an accuracy of 94.9% at the segment level detection. This approach of using MFCC with GMM was also performed in [40] for wheeze detection, by a different group. The data for analysis was recorded from 18 volunteers, with nine of them being asthmatic. 88.1% sensitivity and 99.5% specificity was reported as performance.

A GMM was also used in [90] to perform wheeze segment detection. A total of 24 recordings, with 12 wheezing and 12 normal recordings were obtained from [124] and the ASTRA database CD. The recordings were segmented. 985 wheeze and 1822 normal segments were obtained. Several feature sets were extracted for the classification. The feature sets extracted were based on the Fourier transform, LPC, wavelet transform, and MFCC. The use of an ANN and Vector Quantisation (VQ) as detection techniques was also discussed. The LOOCV technique was used, achieving a sensitivity of 94.6% and a 91.9% specificity, when MFCC was used as a feature with GMM clustering.

Another implementation using, a GMM with MFCC as features, was presented in [87]. The clustering was performed to separate between crackle, wheeze, and stridor sounds. The sound recordings were obtained from an online repository [124]. LOOCV was used with 13 MFCC features. The performance was reported individually as a measure of accuracy of the CV result. The accuracy obtained was 46.1% for the normal data, 98% for crackle, 50% for asthma, and 26.9% for wheeze.

GMM was also used in [51] to separate between crackle and normal recordings. 41 recordings with 14 of them containing crackle sounds were used for classification. Spectral-based features were used for the clustering. The performance claimed was 92.85% sensitivity with 100% specificity.

The study in [57] compared the performance of a GMM and a SVM for the classification of normal and abnormal recordings. An AR model was used as a feature set using LOOCV. The data used was 40 recordings obtained with fourteen SONY ECM-44 BPT microphones, placed on the posterior chest. A best total accuracy of 90% was achieved using a GMM.

A clustering-based classifier similar to a GMM was used in [113] to perform the classification of events based on the underlying pathology. A total of 147 sound events were obtained from [136]. The types of sound observed included normal sounds from varying positions and the sounds of an asthma patient. LPC was used as feature vector for the classification. 42 events were used to find parameters for the clustering-based classifier, based on minimum distance metric; while 105 events were used to test the obtained model. An overall accuracy of 95.24% was achieved as only 5 events were misclassified.

#### Random Forest Based Methods

A Random Forest (RF) was used in [54] to perform wheeze detection. The dataset used was obtained using a Littmann 3M 4000 Electronic Stethoscope on multiple positions on the chest and back of the patient. The signals were obtained from 12 volunteers, and consisted of a total of 24 recordings. 113 wheeze events were annotated in the recordings. The features used for detection were musical features and the spectrogram signature of wheezes, which included the peak selection. The potential wheezes were classified using a RF with the 10-fold CV technique. The performance achieved was 90.9% ± 2% sensitivity and 99.4% ± 1% specificity for the RF wheeze detector. A LRM was also used in the study using the same feature set. The performance achieved for the LRM model was 82.7% ± 2% sensitivity and 98.1% ± 1% specificity.

#### k-Nearest Neighbour Based Methods

The work in [96] used a k-NN method and achieved a 92% sensitivity and a 100% specificity on a test set at the recording level. Classification was performed to differentiate between pathological and normal recordings. Sounds from 65 volunteers recorded using a SONY ECM-44 microphone placed on the posterior chest were used. Data from 40 volunteers was then used as a training set with the LOOCV technique, and the rest was used for test set. AR coefficients were used as a feature set.

The study in [44] used higher order statistics to perform the classification of vesicular, fine and coarse crackle, and monophonic and polyphonic wheeze sounds. The classifier used was a combination of k-NN and NB. The k-NN classifier was used to separate normal, crackle, and wheeze sounds, while two separate NB classifiers were used to further separate fine and coarse crackle and also between monophonic and polyphonic wheeze. A total of 219 events, with 71 normal, 39 each for fine and coarse crackles, and 35 each for monophonic and polyphonic wheezes were used for training. The test was performed using 99 separate events containing 31 normal, 18 each for fine and coarse crackles, and 16 each for monophonic and polyphonic wheezes. 2*^nd^*, 3*^rd^*, and 4*^th^* order cumulants were extracted for each segment and used as features for the classification. A total of 800 features were extracted for each. Feature selection was performed using a Genetic Algorithm (GA) which was found to perform better than Fischer’s Discriminant Ratio (FDR). The classification accuracy obtained was 94.4 ± 1.5% for vesicular sounds, 91.9 ± 2.8% for fine crackles, 90.8 ± 3.2% for coarse crackles, 91.9 ± 2.3% for monophonic wheezes, and 90.3 ± 3.3% for polyphonic wheezes.

Adventitious sound classification was performed using a k-NN classifier in [86]. A total of 585 events, with 264 of them normal, 132 polyphonic wheeze, 93 monophonic wheeze, and 96 stridor events were used for the classification. The recordings were obtained using one SONY ECM-77B microphone. Databases [129, 131, 132] were also used for sounds. LOOCV was used with features extracted based on temporal spectral dominance spectrogram. The performance achieved was 92.4 ± 2.9% overall accuracy.

The same research group as above used a new classification approach which was similar to the k-NN method, called Empirical Classification in [85]. The classifier performed similarly to k-NN, but instead of just checking the local similarity by measuring distance, global similarity was checked based on the variance difference. The feature used for the study was a multi-scale PCA. The classification was performed on data obtained by using one SONY ECM-77B microphone placed on neck. More data were also included from several audio CD companions of books [129, 131, 132]. A total of 689 events, including 130 normal, 413 CAS, and 146 DAS events, were obtained. The performance achieved was 97.3 ± 2.7% for accuracy of classification between normal and CAS and 98.34% between normal and combination of CAS and DAS.

Classification of recordings based on the underlying pathology was performed in [109]. Signals were recorded using two microphones on multiple positions on the chests of 69 volunteers. 28 of the volunteers were obstructive airway disease patients, while 23 of them had restrictive airway disease. At the segment level, LOOCV using a k-NN classifier with an AR model as a feature was performed. A multinomial classifier was employed on the result from each segment to determine the pathology of corresponding respiratory events. The final recording classification was then obtained from voting results of each event. The study achieved an overall accuracy of 71.07% at classifying recordings based on the disease.

#### Hidden Markov Model Based Methods

Hidden Markov Models (HMM) were mainly used by studies from the same research group, as in [43, 52, 60, 79, 93]. The work in [93] used a HMM to perform the classification of abnormal and normal breath sounds. The data used was obtained from 162 volunteers, where 109 of them were patients with emphysema pulmonum. The data was segmented into a total of 1544 events, where 554 of them corresponded to abnormal sounds. The data was recorded using either a condenser or a piezoelectric microphone. LOOCV was carried out and the performance achieved was 93.2% for sensitivity at a 64.8% specificity.

The classification of abnormal respiratory sounds was also the focus in [79], but a new feature was added to improve performance. The duration distribution of noise and abnormal respiratory sounds was used to reduce false alarms caused by noise. The performance achieved by using LOOCV was 88.7% sensitivity and 91.5% specificity for the classification of abnormal versus normal events. Classification of recordings as normal or abnormal was also performed, achieving an 87% sensitivity and an 81% specificity at recognising abnormal recordings.

MFCC were used as features in [43, 52, 60]. An electronic stethoscope was used to obtain data for the analysis. The correlation score with other auscultation points and segments was used as an additional feature to enhance the performance of the HMM in [52]; while [60] used a HMM, which could automatically adapt to different patients by including high-confident previously classified segments to retrain the model. Best sensitivity of 91.10% and specificity of 93.43%, using 8 auscultation points, at the event level, were achieved in [52]; while 89.4% sensitivity and 80.9% specificity, at the event level, were achieved in [60]. The study in [43] combined the timing of occurrence and joint probability of different segments as additional features, achieving a best accuracy of 82.82% at the segment level.

#### Logistic Regression Model Based Methods

A LRM was used in [67] to perform crackle detection. Two recordings were used in the study obtained from [124]. LOOCV was used as a validation method. The performance reported as Matthews Correlation Coefficient (MCC) was 80%. The detection was performed using wavelet, entropy, empirical mode decomposition, Teager energy, and fractal dimension as features. The same group then again employed LRM to perform crackle detection, but using different sets of features [42]. 10-fold CV was performed on 40 recordings obtained using a Littmann 3M 3200 stethoscope from 20 volunteers. The data contained 400 crackle events. The addition of musical features to the feature set resulted in 76 ± 23% sensitivity and 77 ± 22% PPV, at the segment level.

#### Discriminant Analysis Based Methods

A discriminant function was used as a crackle event classification method in [105]. The classification was performed to separate coarse and fine crackles. Recordings from 2 volunteers, with 238 coarse and 153 fine crackles, were used in the analysis. Features were extracted using a wavelet network. The classification model was tested on 158 coarse and 73 fine crackles, and achieved an accuracy of 70% and 84% respectively.

Fischer Discriminant Analysis (FDA) was used as a wheeze and normal sound classifier in [89]. Data taken from 7 volunteers were recorded using fourteen SONY ECM-44 BPT microphones positioned on the chest. The data used for classification was extracted from the recordings in the form of 246 wheeze and 246 normal segments. The feature set was extracted as kurtosis, Renyi Entropy, frequency power ratio, and mean crossing irregularity. The performance reported in the study was a 93.5% accuracy.

A study in [92] performed the classification of squawks and crackles using discrimination analysis. Lacunarity was used as a feature to detect squawk and crackle data obtained from audio CD book companions [129, 132, 134, 135]. The data used was 25 recordings with 136 fine crackles, 93 coarse crackles, and 133 squawk events. The data was separated into 75%-25% train-test set and the process repeated 200 times. The maximum mean accuracy achieved at the segment level was 99.75%.

#### Edge Detection on Spectrogram Image Based Methods

Image processing on the spectrogram of sound recordings was used as a wheeze detection technique in [103]. Wheeze detection was performed on recordings taken from 16 volunteers using one KEC-2738 microphone placed on the neck. Edge detection was applied to obtain horizontal edges which were then processed further to detect wheezes. The study claimed to achieve sensitivity and specificity value above 89%.

### Synthesis of results

The results achieved by the studies reviewed were synthesised as a measure of accuracy range of the algorithms. The synthesis was performed on groups of studies with the same sound type analysed, approach, and level of analysis. The groups considered for the synthesis were wheeze event detection (WED) and wheeze segment detection (WSD), classification between wheeze and other sound at segment (WSC) and event level (WEC), and classification between crackle and other sound at event level (CEC). The studies included in the analysis were articles with relevant information on the dataset size. Performance at the recording level is not analysed further, because for monitoring purposes only segment or event analysis is relevant. Other types were not considered for the synthesis due to the small number of studies having been reported. The summary of accuracy measures synthesised can be seen in Table 6.

Wheeze segment detection reported in [40, 54, 58, 90, 110] achieved an accuracy of 71.2 − 97.9%. At the event level, the achieved accuracy range for wheeze detection by studies in [73, 74, 97, 101, 106] was 79.6 − 100%. Crackle detection at the segment level achieved an accuracy range of 62.7 − 99.8% in studies by [42, 84, 92, 108, 110]. For classification purposes, to differentiate between segments containing wheezes and not, the accuracy achieved by [61, 81, 89, 99, 111] was 90.5 − 96%. For classification between wheeze event and other types of sound, the accuracy of studies in [41, 46, 55, 59, 63, 65, 69, 83, 86, 91, 94, 98] was between 75.78 − 100%. Crackle event classification, as reported in [46, 55, 62, 91], achieved an 89 − 98.15% accuracy range. Based on the accuracy range reported, both wheeze and crackle sound automatic analyses showed that high agreement with the expert can be achieved under controlled conditions.

## Discussion

The systematic review of algorithm development for adventitious sounds analysis is discussed in this section. This discussion is followed by a summary of the main findings, challenges, and future work in developing automatic adventitious respiratory sound analysis methods. Limitations and conclusions of this systematic review are finally given.

### Development of automated adventitious sound analysis algorithms

There are two approaches in automated adventitious sound analysis, as can be seen in Table 3. The first approach is to perform detection, while classification is the second approach. The difference between these two approaches is on the purpose of analysis. The purpose of the detection approach is to determine whether or not adventitious sounds exist in a sound signal. The purpose of the classification approach is to determine if a certain sound signal belongs to a certain class.

For an automated symptoms monitoring and management tool, real time adventitious sound monitoring may be needed. The development of real-time processing could allow for the timely identification of diseases, as well as changes in their severity. This functionality is important. For example, for the early detection and prevention of exacerbations. A detection approach could be used directly as it generally works at the segment level, allowing for the development of real-time processing in a straight-forward manner. For a classification approach to be used for monitoring, each breath cycle needs to be automatically segmented first, and isolated events need to be extracted. It is worth taking into account, however, that both approaches can be challenging in real life scenarios—as opposed to the controlled conditions normally used to extract data for algorithm development—due to the presence of strong acoustic artefacts that will corrupt the signal of interest [144].

Different sound types are related to different diagnoses. In the papers reviewed, a focus was given to wheeze and crackle analysis. A limited number of references used egophony, squawk, as well as pleural rub sounds in their analyses. It is also possible to perform analysis on how the adventitious sounds were generated, such as in [70].

Stethoscopes and microphones were generally used as the instrumentation to collect data for analysis. Several references also used data acquired from databases, which were mostly recorded using a digital stethoscope. Using a stethoscope for monitoring purposes may not be practical, as this is not a viable solution for continuous sensing. Using a microphone attached to the body, as in several references, would be a more desired approach, since this could potentially be done without disrupting the patient’s normal activities.

The number of sensors as well as positioning of those sensors in the reviewed literature, was also provided (in Table 4). The works which used stethoscopes as the instrument to collect data mostly performed data collection from multiple positions on the body. For a device to be non-intrusive and easy to use, it is important that the analysis is performed on a data obtained from a single location. This will also greatly increase the probabilities of patient’s compliance.

The positions which are used most often to place the sensors in the reviewed literature were the anterior and posterior chest wall. These locations are used in the conventional auscultation method. However, as discussed in the previous sections, the chest wall acts as a low-pass filter, which limits the frequency range of the sounds heard. Another problem is that sounds heard from the chest are limited at the expiration phase. This will reduce the amount of information which can be used for analysis. Collecting data from the trachea may, in some cases, be a better option as the dynamic range is wider, the sounds generated contain energy at higher frequencies, and the sound intensity is louder.

Obtaining data from different patients is also important, to be able to generalise the algorithms developed. Analysis performed using training and test sets from the same patients may cause an algorithm to be patient specific and reduce the generality of the model. Obtaining more data may also give more insight into the relevance or importance of the newly found features. It may also be useful to carry out research on whether the characteristics of adventitious sounds are, for example, population or disease severity specific.

Machine learning techniques have gained a lot of interest and, as seen in the previous section, are used by most reported works. SVM and ANN variants were mostly used as classification methods. In these, it is important to find features that can differentiate between normal and abnormal segments for the detection or classification method to perform well. The complexity of a method is not only influenced by the type of detection or classification method used, but also by the complexity of the feature extraction. Using a high number of features may cause the detection or classification to over-fit the current data, resulting in the method not being generalisable in new data.

### Challenges and future works

Adventitious sounds monitoring is an integral part of the management of diseases such as asthma and COPD. Regular monitoring of lung function, and symptoms such as wheezes, crackles, cough, and breathlessness are needed for disease management, and could potentially be used for exacerbation prediction. However, continuous monitoring and management of adventitious sounds are challenging tasks to accomplish. Significant research is still needed to overcome these challenges. The focus of future work could be divided into several main categories, as follows.

Algorithms for adventitious sound analysis could be improved further. Algorithms developed need to have a high accuracy to detect or classify adventitious sounds. More research could be carried out to find new features with high correlation with adventitious sounds characteristics; aiming to achieve high performance measures, even in real life scenarios in which the signals are going to be far more corrupted than those used in controlled experiments for algorithm development. Better signal to noise ratio could also improve analysis performance.

Most literature reviewed reported a high performance measure, but many of the works reported performance on CV sets instead of separate test sets. The problem stated in most published literature was lack of data, which caused LOOCV to be often used as a validation method. Performance measures obtained from cross-validation, especially those used for parameter tuning and model selection, can introduce high variance thus making the model unreliable [145–147]. In future works, particularly for machine learning based algorithms, it is recommended to report performance on a separate test set instead of a CV set. A separate test set contains new information not seen in model training and parameter optimisation and can give a more objective performance measure which will prevent over-fitting problems.

Increasing the performance of algorithms for adventitious sound analysis is important to assure the validity of the systems developed. Algorithm validity is important because doctors and patients tend to underestimate the severity of present symptoms [26]. With accurate detection of symptoms, the device developed could be used as a reference to the required treatment based on actual severity. This will ensure that the disease is properly treated and managed.

Another important research focus should be on making a device that can be used by patients. There are several devices available to perform monitoring of symptoms and lung function at home, but these are mostly complex and large [25]. An optimum device should be portable and easy to use so that patient compliance in self-monitoring can be assured. In some cases, symptoms most often occur at night. Hence, an automated device that can continuously monitor symptoms without the need of expert interference is necessary. The size, number, and positioning of the sensors will also influence the usability. More complex systems will be harder to use, and hence the intended purpose may not be achieved. Newly developed devices also need to be non-intrusive so that they can be used without causing a disruption to daily activities.

Using as foundations the detection and classification of adventitious sounds algorithms, new ones can be further developed to perform exacerbation prediction. Exacerbation prevention can help patients avoid worsening of conditions and adverse effects on the respiratory system.

One of the main drawbacks of conventional auscultation is that it cannot be performed frequently [25]. As symptoms such as wheeze generally occur at night, an ideal device will be able to monitor these symptoms during the night. Power consumption issues need to be taken into account in future works, as well as the storage capacity in the device. The data could be processed so that only the results of symptoms monitoring are stored, or if possible, raw data can be saved for future reference.

### Study limitations

The metrics used for this systematic review have been measure and comparison of accuracy. The main limitation of this study at the outcome level is that the data used in each published reference was different. Each work performed analysis on data from a different population and obtained with different collection methods. A standard validation and data management method has not been established; different methods were used across studies. Outcome measure definition also varied between different works. At the review level, the main limitation is the difficulty in assessing the quality of the different studies, as there is no standardised criterion yet.

## Conclusion

This systematic review provided an introduction to the types of respiratory sounds and their analysis, with a focus on automatic adventitious sound detection or classification for disease monitoring and management.

The characteristics of normal and abnormal breath sounds, specifically adventitious sounds, were discussed. Several types of normal breath sounds based on their location were summarised. Adventitious sound definitions and characteristics were also reviewed. Diseases related to some of the adventitious sounds were briefly introduced.

References to algorithms development for adventitious sound detection or classification were also reviewed. For each paper the type of sound, approach, level of analysis, instrumentation, sensor number and positioning, total amount of data, features, methods, and performance were provided and summarised.

Overall, based on the accuracy metric used in this systematic review, algorithms for automatic detection or classification of adventitious sounds achieved high agreement with the expert under controlled conditions. This makes automated adventitious sounds detection or classification a promising solution to overcome the limitations of conventional auscultation. Recommendations for future research and development would be:

- To pay increased attention to how to split the data for algorithm development in order to avoid under-fitting, over-fitting or patient specific results.
- To focus on increasing performance, ensuring usability and availability of sensors.
- To add functionality leading, for example, to exacerbations prediction.
- To carry out algorithms’ validation in real life use scenarios.

## Supporting information

S1 File PRISMA checklist. PRISMA Checklist document showing pages where items are reported.
(PDF)
